# Two Novel Small Molecule Donors and the Applications in Bulk-Heterojunction Solar Cells

**DOI:** 10.3389/fchem.2018.00260

**Published:** 2018-07-02

**Authors:** Xin Qi, Yuan-Chih Lo, Yifan Zhao, Liyang Xuan, Hao-Chun Ting, Ken-Tsung Wong, Mostafizur Rahaman, Zhijian Chen, Lixin Xiao, Bo Qu

**Affiliations:** ^1^State Key Laboratory for Artificial Microstructures and Mesoscopic Physics, Department of Physics, Peking University, Beijing, China; ^2^Department of Chemistry, National Taiwan University, Taipei, Taiwan; ^3^Department of Chemistry, King Saud University, Riyadh, Saudi Arabia; ^4^New Display Device and System Integration Collaborative Innovation Center of the West Coast of the Taiwan Strait, Fuzhou, China

**Keywords:** bulk-heterojunction, small molecule, donor, solar cell, ditolylaminothienyl, quinoxaline

## Abstract

Two novel small molecules **DTRDTQX** and **DTIDTQX**, based on ditolylaminothienyl group as donor moiety and quinoxaline as middle acceptor moiety with different terminal acceptor groups were synthesized and characterized in this work. In order to study the photovoltaic properties of **DTRDTQX** and **DTIDTQX**, bulk-heterojunction solar cells with the configuration of FTO/c-TiO_2_/**DTRDTQX**(or **DTIDTQX**):C_70_/MoO_3_/Ag were fabricated, in which **DTRDTQX** and **DTIDTQX** acted as the donors and neat C_70_ as the acceptor. When the weight ratio of **DTRDTQX**:C_70_ reached 1:2 and the active layer was annealed at 100°C, the optimal device was realized with the power conversion efficiency (PCE) of 1.44%. As to **DTIDTQX**:C_70_-based devices, the highest PCE of 1.70% was achieved with the optimal blend ratio (**DTIDTQX**:C_70_ = 1:2) and 100°C thermal annealing treatment. All the experimental data indicated that **DTRDTQX** and **DTIDTQX** could be employed as potential donor candidates for organic solar cell applications.

## Introduction

Recently, organic solar cells (OSCs) based on bulk-heterojunction structure have attracted much attention due to the distinctive characteristics of low cost, easy fabrication, flexibility and light weight, etc. (Gustafsson et al., 1992; Shaheen et al., 2001; Chen and Cao, 2009). Compared with polymers employed in solar cells, small molecule donors have the advantage of less batch-to-batch variation, well-defined molecular structure, easier purification, etc. (You et al., 2013; Chen et al., 2014, 2015; He et al., 2015; Zhou et al., 2015). Therefore, much work focused on small molecule donors and the photovoltaic performance of OSCs was improved accordingly (Sun et al., 2011; Liu et al., 2013; Love et al., 2013; Coughlin et al., 2014). In general, the active layers of the solar cells consisted of small molecule donors and fullerene/fullerene derivative acceptors (Chen et al., 2012; Huang et al., 2016). In order to optimize the photovoltaic characteristics of OSCs, narrow band-gap and deep highest occupied molecular orbital (HOMO) of small molecule donors should be considered, which resulted in broad absorption and high open-circuit voltage (V_oc_) of devices. Then, various small molecules composed of electron rich moieties (donor, “D”) and electron deficient moieties (acceptor, “A”), have been reported with the molecular configuration such as D-A (Roquet et al., 2006), A-D-A (Schulze et al., 2006), D-A-A (Lin et al., 2011) and D-A-D conjugated structures. In this regard, the HOMO and lowest unoccupied molecular orbital (LUMO) of the small molecules were effectively tuned, mainly due to the intramolecular charge transfer (ICT) between donors and acceptors (Zhang et al., 2011).

**Figure 1:**
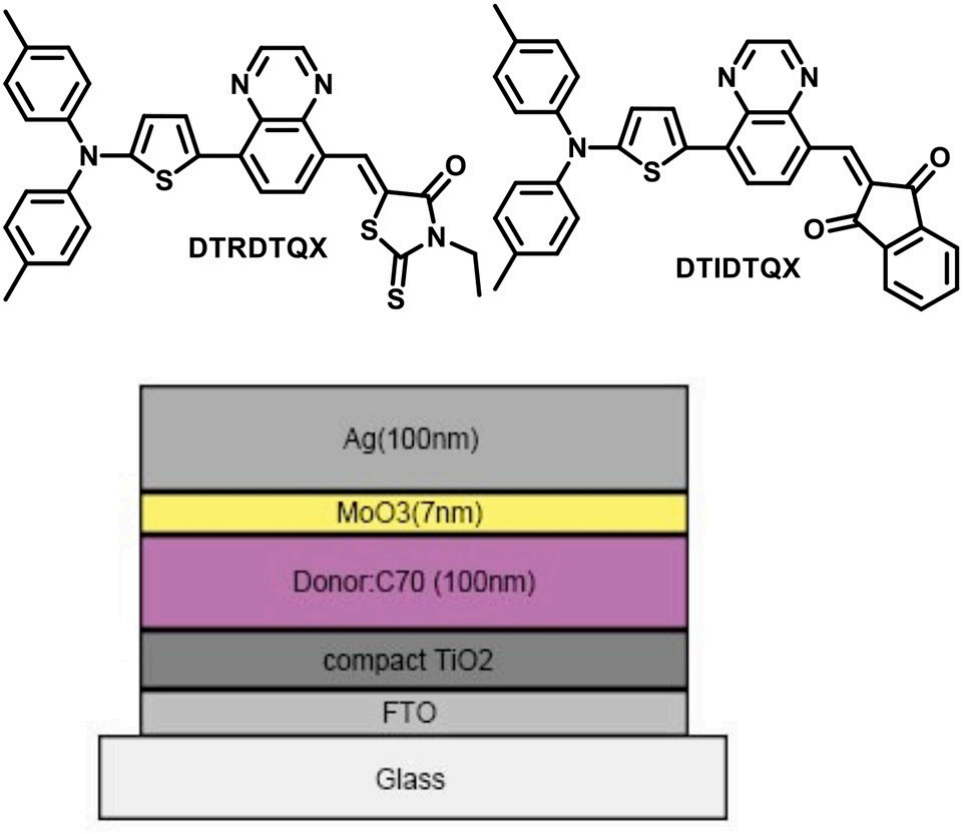
Molecular structures of **DTRDTQX** and **DTIDTQX**, and the configuration of OSCs.

Herein, the photovoltaic properties of two novel small molecule donors (named **DTRDTQX** and **DTIDTQX**, Figure 1) based on D-A-A structure were studied in this work. **DTIDTQX** or **DTRDTQX** consisted of ditolylaminothienyl group as the donor moiety, quinoxaline as middle acceptor moiety with different terminal acceptor groups such as 1,3-indandione or 3-ethylrhodanine, respectively. To investigate the photovoltaic properties of the small molecules, bulk-heterojunction (BHJ) solar cells based on **DTRDTQX** or **DTIDTQX** as the donor together with C_70_ as the acceptor were fabricated and the optimal cells showed PCE of 1.44 and 1.70%, respectively.

## Experimental

### Materials and characterization

All materials in this work were purchased commercially, except for the tailor made **DTRDTQX** and **DTIDTQX** donors. The commercial materials were used without further purification.

**Scheme 1:**
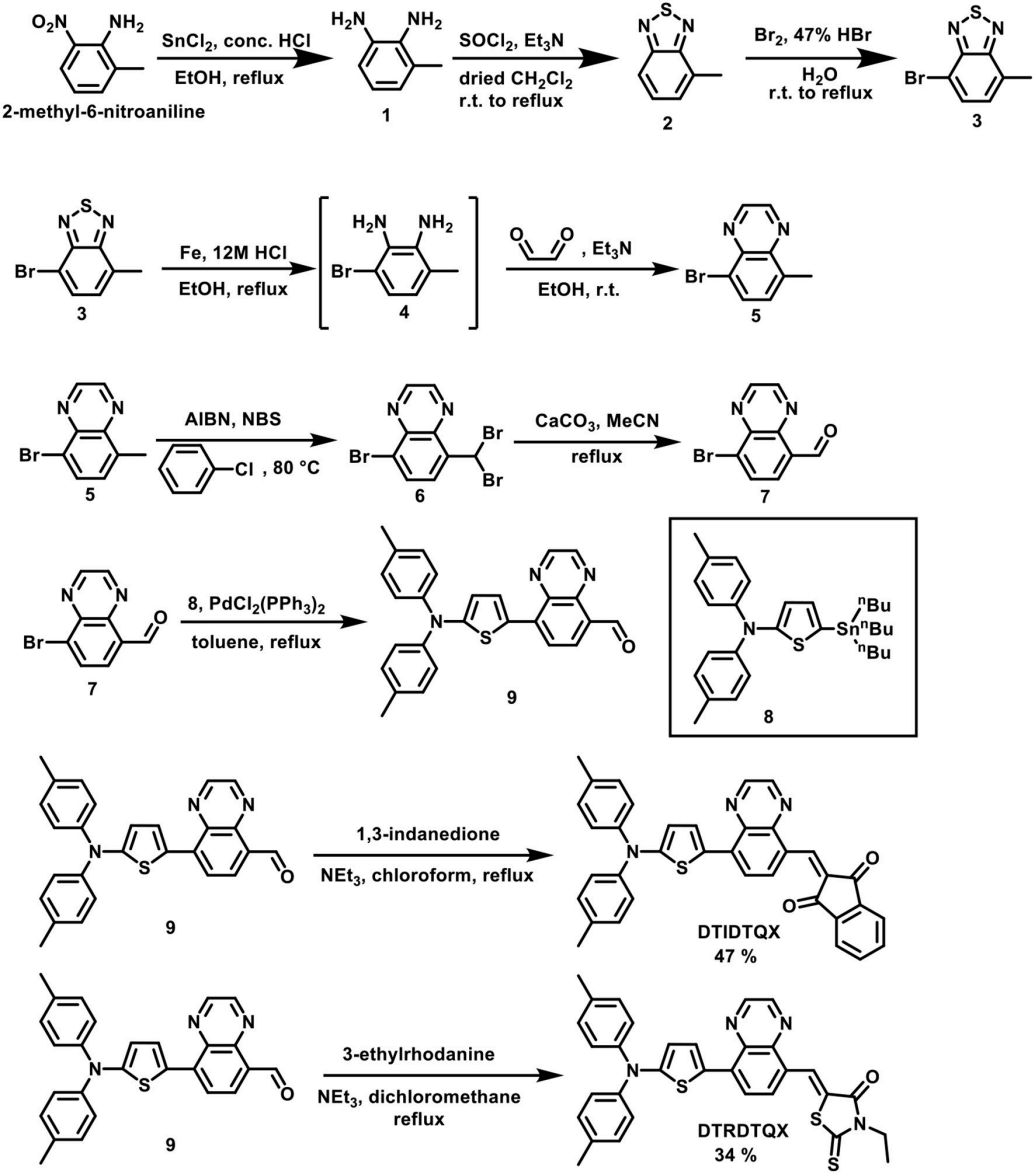
Synthetic route of **DTIDTQX** and **DTRDTQX**.

Scheme 1 depicts the synthesis of **DTIDTQX** and **DTRDTQX**. By following the protocols established by Krebs et al. (Jorgensen and Krebs, 2005) and Janssen et al. (Bijleveld et al., 2009), we could get 4-bromo-7-methyl-2,1,3-benzo-thiadiazole (**3**). Then the hetereocyclic **3** was converted to diamine intermediate **4** by treating Fe/HCl, which was then followed by condensation with glyoxal to afford 5-bromo-8-methylquinoxaline (**5**) without further purification. The 8-bromoquinoxaline-5-carbaldehyde (**7**) was synthesized by benzylic bromination with N-bromosuccinimide (NBS) initiated by azobisisobutyronitrile (AIBN) and followed by hydrolysis with CaCO_3_ in H_2_O/acetonitrile (Lin et al., 2011). Aldehyde **7** was reacted with N,N-di-p-tolyl-5-(tri-n-butylstannyl)-thiophen-2-amine (**8**) through Stille coupling reaction and gave key intermediate **9**. Finally, the condensation of **9** with 1,3-indandione and 3-ethylrhodanine via Knöevenagel reaction afforded **DTIDTQX** and **DTRDTQX**, respectively. The absorption spectra were measured with JASCO V-670 spectrophotometer. Themogravimetric analysis (TGA) was determined on a TA Instruments Model TGA Q500 V20.13 (build 39) with a heating rate of 10°C/min. Differential Scanning Calorimeter (DSC) was carried out at a heating rate of 10°C/min on a TA Instruments Model DSC Q100 V9.9 (build 303). The thickness of the films was evaluated using a surface profilometer. The electrochemical cyclic voltammetry (CV) was recorded by a CHI619B potentiostat with glassy carbon electrode, Pt wire and Ag/AgCl which were used as the working electrode, counter electrode, and reference electrode, respectively, further calibrated with the ferrocene/ferrocenium (Fc/Fc^+^) redox couple. The oxidation waves were recorded in CH_2_Cl_2_ (for 1.0 mM) with 0.1 M tetrabutylammonium hexafluorophosphate (^n^BuNPF_6_) as supporting electrolyte, while reductive waves were recorded in THF (for 1.0 mM) with 0.1 M tetrabutylammonium perchlorate (^n^BuNClO_4_) as supporting electrolyte.

### Solar cell fabrication and characterization

In order to investigate the photovoltaic properties of **DTRDTQX** and **DTIDTQX**, the OSCs with the configuration of FTO/c-TiO_2_/**DTRDTQX**(or **DTIDTQX**):C_70_/MoO_3_/Ag were fabricated as shown in Figure 1. The compact TiO_2_ layer in OSCs acted as the electron transporting layer (Heo et al., 2015) and MoO_3_ as the hole buffer layer. As to the photoactive layers, **DTRDTQX** and **DTIDTQX** served as the donors and C_70_ as the acceptor, respectively. The FTO cathode was pre-cleaned in an ultrasonic cleaner with deionized water, acetone and alcohol for 15 min respectively and then treated with oxygen plasma for 15 min. The TiO_2_ films were fabricated according to the literatures (Kim et al., 2012; Zhang et al., 2016) and sintered at 500°C for 15 min in a muffle furnace. And then, the TiO_2_ films were naturally cooled to room temperature. Blended solutions (total concentration: 20 mg/ml) of **DTRDTQX**(or **DTIDTQX**):C_70_ in *ortho*-dichlorobenzene (oDCB) were spin-coated (700 rpm, 18 s) onto FTO/TiO_2_ substrates in a glove box and then thermal annealed at 100°C or 150°C. The effect of thermal annealing on the photovoltaic properties of the active layers was also studied in this work. Finally, 7 nm MoO_3_ buffer layers and 100 nm Ag anodes were thermal evaporated successively below 10^−6^ Torr. The photovoltaic performance of the OSCs were evaluated by current density-bias voltage (J-V) measurement (using a Keithley 2400 source meter) under AM 1.5G simulated solar illumination (Newport model 94021A, 100 mW cm^−2^).

## Results and discussion

### Thermal property

**Figure 2:**
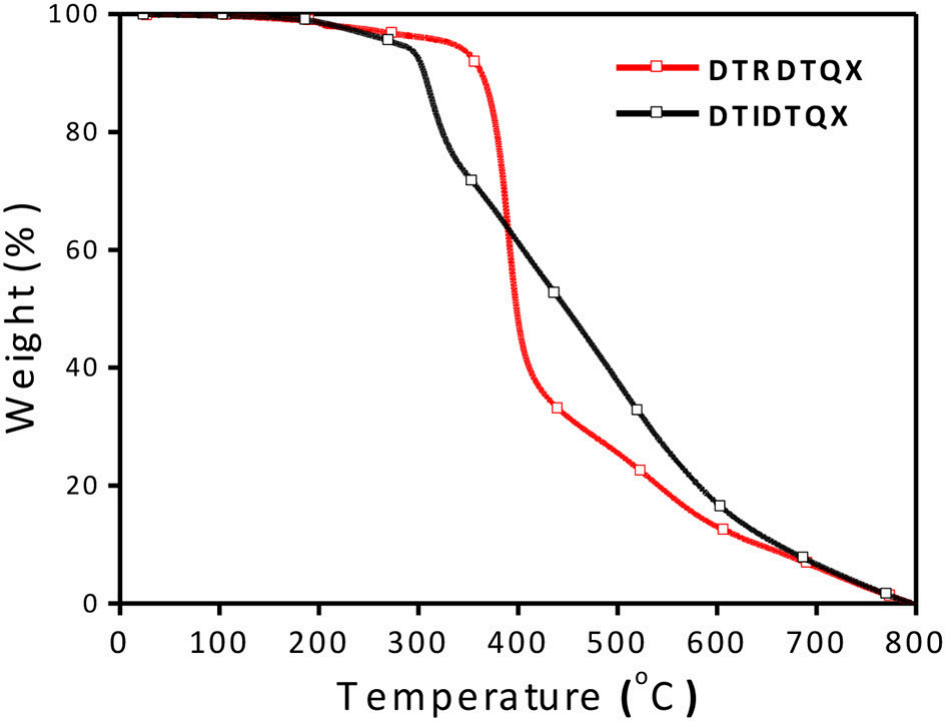
TGA diagrams of **DTRDTQX** (up) and **DTIDTQX** (down).

**Figure 3:**
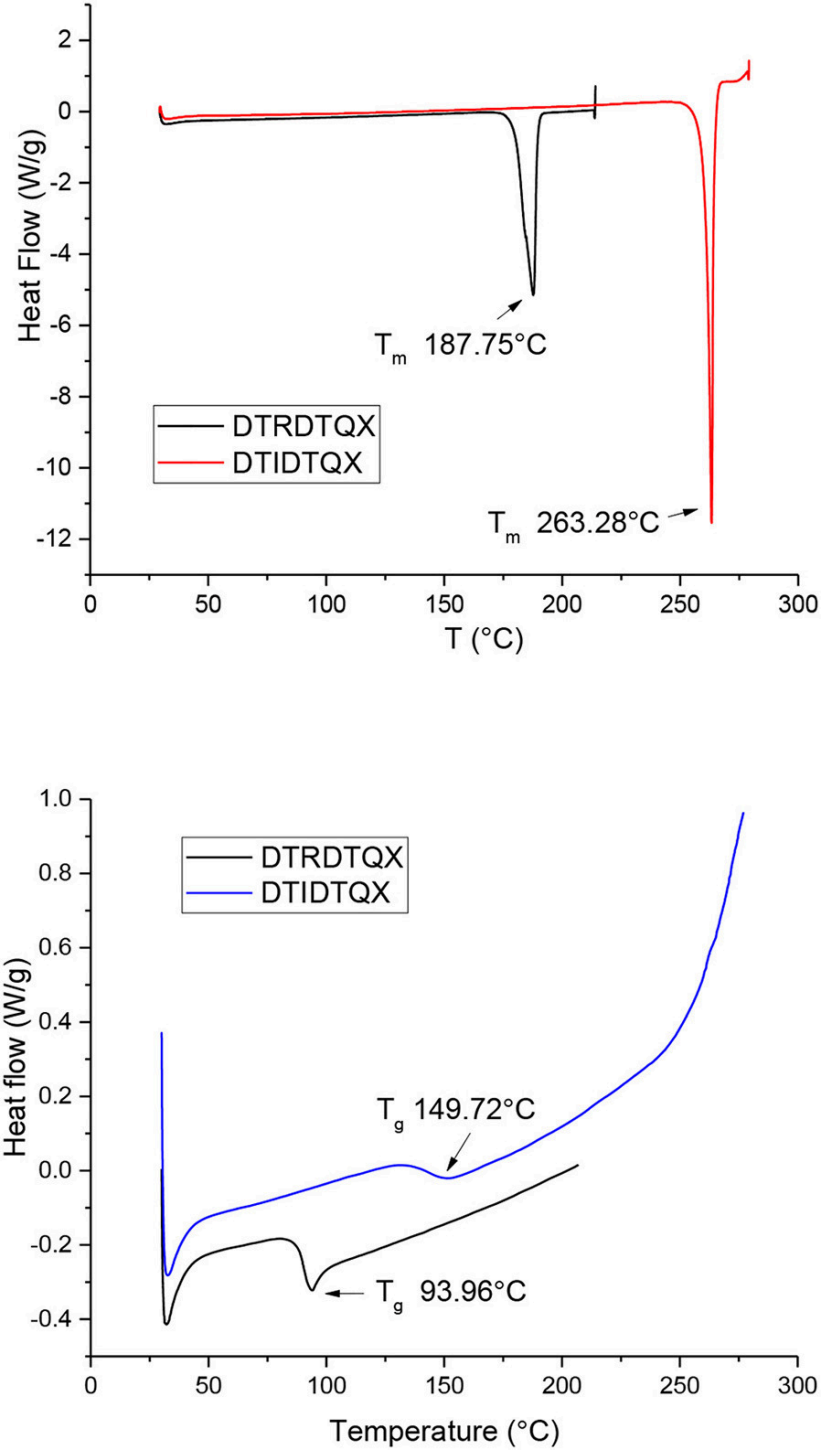
Differential scanning calorimetry measurements of **DTRDTQX** and **DTIDTQX**.

Thermal properties of the two small molecules were investigated by TGA measurement as shown in Figure 2 and the thermal decomposition temperatures (T_d_, 5% weight loss) were evaluated to be 362°C and 312°C for DTRDTQX and DTIDTQX respectively, indicating the good thermal stability of the small molecules. According to the DSC plots shown in Figure 3, the melting temperatures (T_m_) were evaluated to be 187.8°C and 263.3°C for DTRDTQX and DTIDTQX, respectively. Moreover, the glass transition temperatures (T_g_) were measured to be 94.0°C and 149.7°C for DTRDTQX and DTIDTQX, respectively. Therefore, both DTRDTQX and DTIDTQX were stable donors for OSCs due to their decent thermal stability.

### Absorption properties

**Figure 4:**
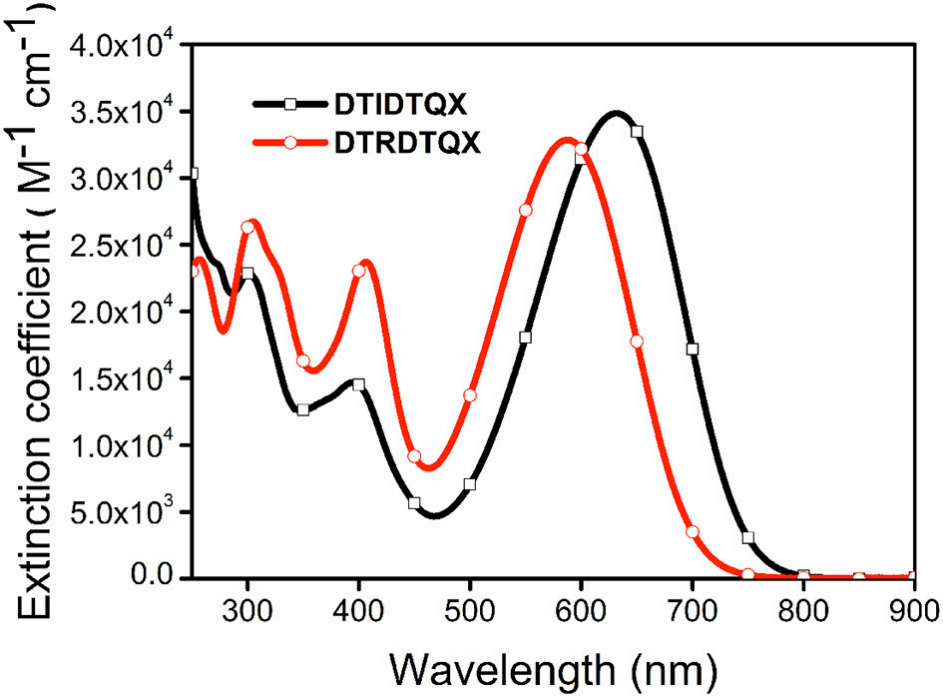
UV-Vis absorption spectra of **DTIDTQX** and **DTRDTQX** in CH_2_Cl_2_.

The UV-Vis absorption of **DTIDTQX** and **DTRDTQX** in CH_2_Cl_2_ were shown in Figure 4 and the corresponding data were summarized in Table 1. The compounds showed broad band absorption from 480 to 750 nm with high extinction coefficient (3.3–3.5 × 10^4^ M^−1^cm^−1^) in the visible range (450–700 nm). **DTIDTQX** absorbed longer wavelength than **DTRDTQX** (631 vs. 588 nm), mainly due to the stronger electron withdrawing ability of 1,3-indanedione group than that of N-ethylrhodanine group.

**Table 1:** Physical properties of **DTIDTQX** and **DTRDTQX**.

**Compounds**	**λ_abs_ solution (nm)^a^ (ε, M^−1^cm^−1^)**	**Δ*E*^opt^ sol. (eV)^a^**	**${\text{E}}_{\text{onset}}^{\text{ox}}$ (V)^b^**	**${\text{E}}_{\text{onset}}^{\text{red}}$ (V)^b^**	**Δ*E*^CV^ (eV)**	**HOMO (eV)^b^**	**LUMO (eV)^b^**	**Td (°C)**
DTIDTQX	631 (34,900)	1.97	0.23	−1.14	1.37	−5.33	−3.96	312
DTRDTQX	588 (32,800)	2.11	0.19	−1.32	1.70	−5.29	−3.59	361

a *Measured in CH_2_Cl_2_ solution (10^−5^ M) and the value was estimated from the onset*.

b *Estimated from the HOMO (−5.1 eV) (Cardona et al., 2011) of Fc^+^/Fc as reference*.

*^c^ Temperature corresponding to 5% weight loss obtained from TGA analysis*.

### Electrochemical properties

**Figure 5:**
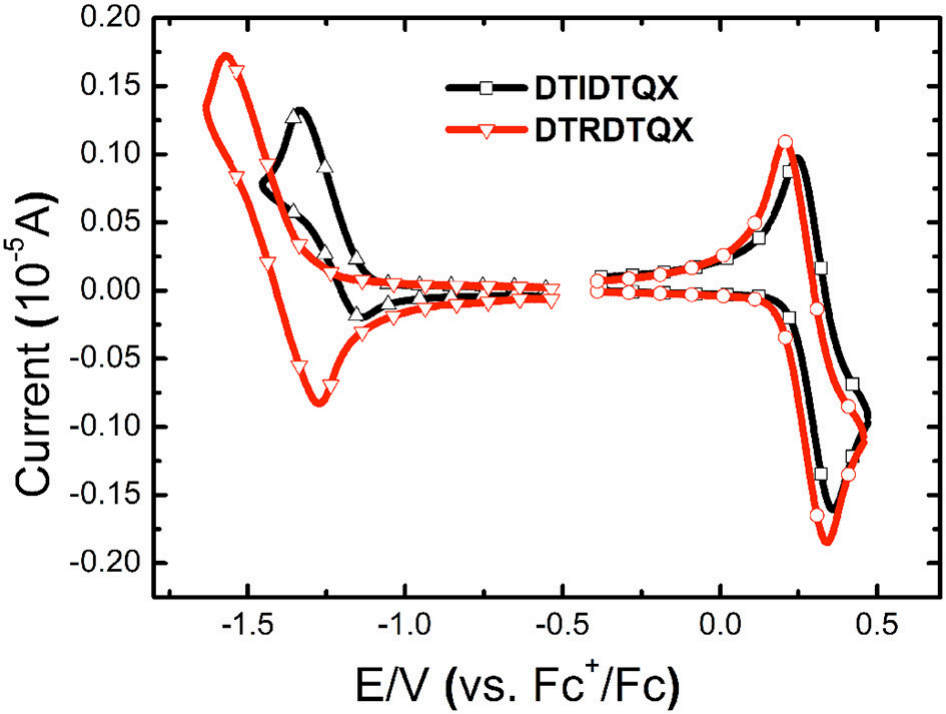
Cyclic voltammograms of **DTIDTQX** and **DTRDTQX**.

**Figure 6:**
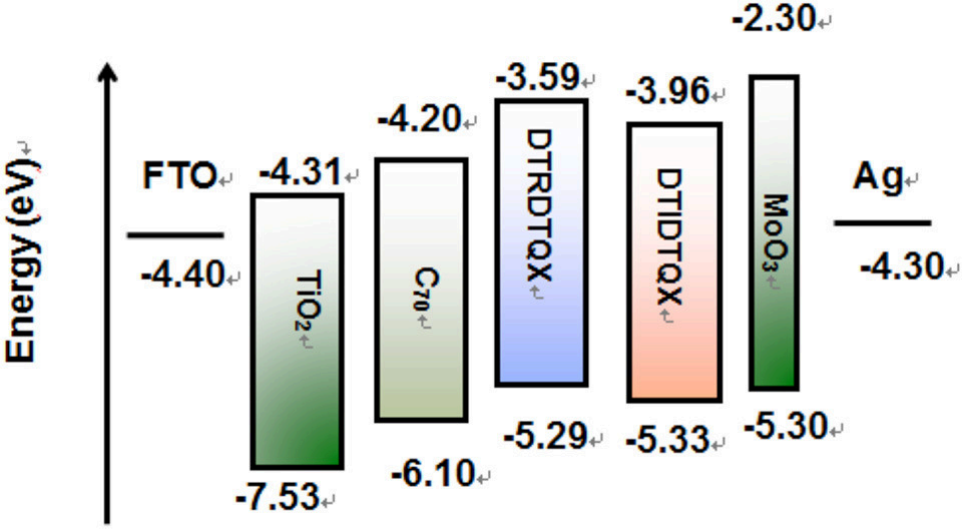
The energy levels of the materials used in the OSCs (Lau et al., 2013; Zhang et al., 2013; Xiao et al., 2016).

The electrochemical properties of **DTRDTQX** and **DTIDTQX** were studied with cyclic voltammetry (CV) as shown in Figure 5. In addition, the energy levels as well as the band gaps of **DTRDTQX** and **DTIDTQX** were summarized in Table 1. With the oxidation and reduction potentials recorded, the HOMO and LUMO levels of the two materials could be calculated (HOMO = −5.1 eV – ${\text{E}}_{\text{onset}}^{\text{ox}}$, LUMO = −5.1 eV – ${\text{E}}_{\text{onset}}^{\text{red}}$), which were −5.33 eV, −3.96 eV for **DTIDTQX** and −5.29 eV, −3.59 eV for **DTRDTQX** respectively. Interestingly, the HOMO and LUMO levels of **DTIDTQX** were both deeper than those of **DTRDTQX**. The phenomenon implied that the electron withdrawing ability of 1,3-indanedione group was stronger than that of N-ethylrhodanine group, which was consistent with the observation of UV-Vis absorption. The energy levels of the materials used in the OSCs were depicted in Figure 6. The large gap between the low-lying HOMO level (−5.33 eV) of **DTIDTQX** and LUMO (−4.20 eV) of C_70_ was evaluated to be 1.13 eV, which resulted in the large V_oc_ (0.71 V) of the optimal **DTIDTQX**-based OSCs in this work. Furthermore, the electrochemical energy band gap (Δ*E*^CV^) of **DTIDTQX** was 0.33 eV lower than that of **DTRDTQX** and strong absorption of **DTIDTQX** active layer in red region could be realized, which was matched well with the UV-Vis absorption spectrum shown in Figure 4. Therefore, the light-harvesting capability as well as the photovoltaic performance of **DTIDTQX-**based devices could be superior to that of **DTRDTQX**-based counterparts, which will be discussed further in following.

### Photovoltaic properties

**Figure 7:**
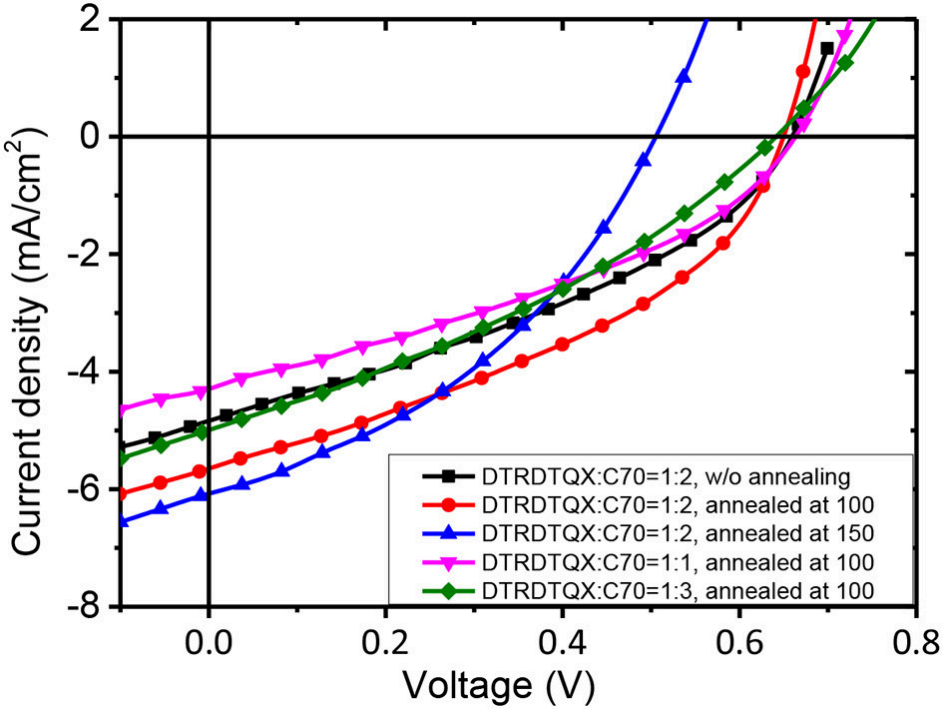
The J-V curves of **DTRDTQX**-based devices.

**Figure 8:**
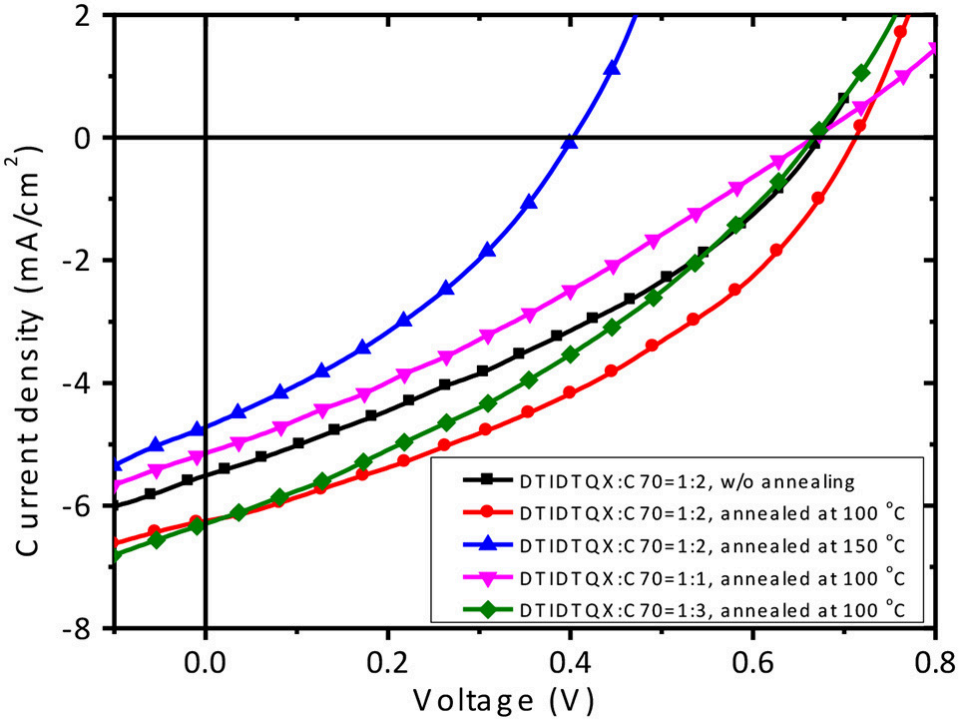
The J-V curves of **DTIDTQX**-based devices.

To study the photovoltaic properties of the small molecules, OSCs with the structure of FTO/c-TiO_2_/donor:C_70_/MoO_3_/Ag were fabricated. The weight ratios of **DTRDTQX**:C_70_ and **DTIDTQX**:C_70_ varied from 1:1 to 1:3 and the corresponding J-V curves of the OSCs were shown in Figures 7, 8. All the photovoltaic data of OSCs were summarized in Table 2. When the weight ratio of **DTRDTQX**:C_70_ reached 1:2 and the photoactive layer was thermal annealed at 100°C, the best **DTRDTQX**-based OSC was realized with the short-circuit current density (J_sc_) and PCE of 5.66 mA/cm^2^ and 1.44%, respectively. The champion **DTRDTQX**-based OSC exhibited almost the same open-circuit voltage (V_oc_) of ~0.65 V as other OSCs with different weight ratios (1:1 and 1:3) of **DTRDTQX**:C_70_. Moreover, for the devices based on **DTRDTQX**:C_70_ with the weight ratios of 1:1 and 1:3, the decreased J_sc_ was mainly ascribed to the imbalanced electron and hole diffusion in the OSCs (Kim et al., 2009). The photovoltaic data in Table 2 implied that the weight ratio (**DTRDTQX**:C_70_) of 1:2 was advantageous to the photovoltaic performance of **DTRDTQX**:C_70_-based OSCs. The photovoltaic properties of **DTRDTQX**:C_70_(1:2)-based OSCs with 150°C thermal annealing and without thermal annealing were also studied and compared. The V_oc_ and PCE of the OSC with 150°C thermal annealing were decreased to 0.51 V and 1.19%, respectively. As to the OSC without thermal annealing, the PCE was decreased to 1.14% and V_oc_ (~0.66V) was almost unchanged compared with the champion **DTRDTQX**-based OSC. Therefore, 100°C thermal annealing treatment was necessary for the reasonable photovoltaic performance of **DTRDTQX**:C_70_(1:2)-based OSCs according to the experimental data.

**Table 2:** Photovoltaic data of the OSCs.

**DTRDTQX: C_70_**	**Thermal annealing**	**V_oc_ (V)**	**J_sc_ (mA/cm^2^)**	**FF**	**PCE (%)**
1:1	100°C	0.66	4.27	0.36	1.01
1:2	100°C	0.65	5.66	0.39	1.44
1:3	100°C	0.64	5.00	0.33	1.05
1:2	150°C	0.51	6.09	0.38	1.19
1:2	w/o	0.66	4.86	0.36	1.14
**DTIDTQX: C_70_**					
1:1	100°C	0.67	5.13	0.30	1.02
1:2	100°C	0.71	6.24	0.38	1.70
1:3	100°C	0.67	6.31	0.34	1.43
1:2	150°C	0.40	4.71	0.35	0.66
1:2	w/o	0.67	5.51	0.34	1.26

As to **DTIDTQX**-based OSCs, the photovoltaic performance was modulated by the weight ratios of **DTIDTQX**:C_70_ from 1:1 to 1:3. When the blend ratio of **DTIDTQX**:C_70_ reached 1:2, the best **DTIDTQX**-based OSC was realized as shown in Table 2. The V_oc_, J_sc_, FF, and PCE of the champion device were 0.71V, 6.24 mA/cm^2^, 0.38 and 1.70%, respectively. It was worthy to note that the V_oc_ of **DTIDTQX**:C_70_(1:2)-OSC was 0.06 V higher than that of **DTRDTQX**:C_70_(1:2)-OSC, mainly due to the low-lying HOMO (−5.33 eV) of **DTIDTQX** as shown in Figure 6. Moreover, the J_sc_ and PCE of **DTIDTQX**:C_70_(1:2)-OSC were both higher than those of **DTRDTQX**:C_70_(1:2)-OSC. Therefore, the photovoltaic properties of **DTIDTQX**-based devices were superior to those of **DTRDTQX**-based counterparts, which was mainly ascribed to the narrow band gap (~1.37 eV) of **DTIDTQX** and the consequent effective absorption in solar spectrum. The photovoltaic performance of **DTIDTQX**:C_70_(1:2)-OSC was deteriorated when the active layer was treated with 150°C thermal annealing as shown in Table 2. And when **DTIDTQX**:C_70_(1:2)-OSC was fabricated without thermal annealing, the PCE decreased to 1.26%. Therefore, 100°C thermal annealing was favorable to **DTIDTQX**:C_70_(1:2)-OSC and a decent PCE of 1.70% was obtained accordingly. However, the FF values of the OSCs were relatively low in this work and much work should be required to further increase FF as well as PCE of the OSCs, such as inserting buffer layers (Ji et al., 2016; Li et al., 2016; Mbuyise et al., 2016), introducing optical spacers (Ben Dkhil et al., 2014), employing solvent annealing (Sun et al., 2014; Li et al., 2015), chemical treatments (Bai et al., 2015), etc.

**Figure 9:**
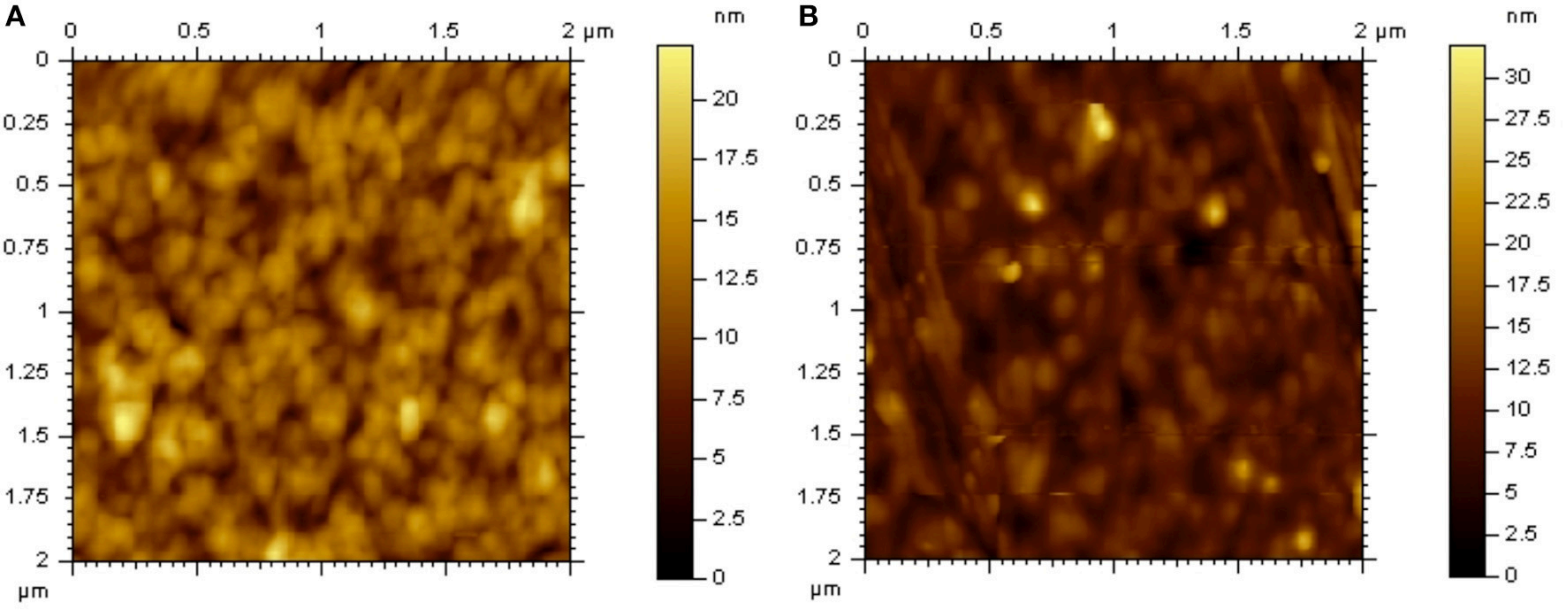
AFM images of **(A) DTRDTQX**:C_70_(1:2) and **(B) DTIDTQX**:C_70_(1:2) films.

**Figure 10:**
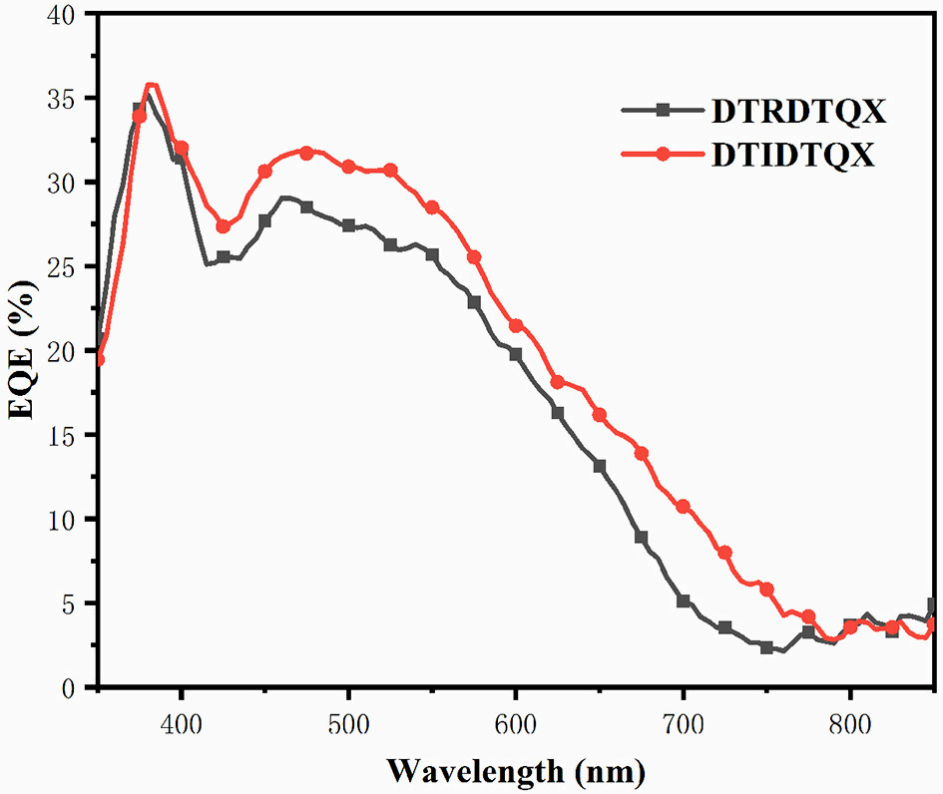
The EQE spectra of **DTRDTQX** and **DTIDTQX**-based devices.

The morphology of **DTRDTQX**:C_70_(1:2) and **DTIDTQX**:C_70_(1:2) films was studied by atomic force microscopy (AFM) (Agilent Series 5500) as shown in Figure 9. The root-mean-square roughness (RMS) of **DTIDTQX**:C70 (1:2) film was 2.94 nm, which was a little higher than that of **DTRDTQX**:C70 (1:2) film (2.58 nm), The relatively low RMS of **DTRDTQX**:C_70_(1:2) and **DTIDTQX**:C_70_(1:2) facilitated the reasonable photovoltaic performance of the corresponding devices. Besides, the external quantum efficiency (EQE) spectra of the champion devices were measured with a lock-in amplifier (model SR830 DSP) as shown in Figure 10. The EQE of **DTIDTQX**-based device was higher than that of **DTRDTQX**-based counterpart and the integrated photocurrent was 5.47 and 4.71 mA/cm^2^, respectively, which was consistent with the photovoltaic properties of the corresponding OSCs. In order to further study the charge transporting properties of the p-type small molecules, hole mobility was measured by using the space-charge-limited current (SCLC) method and the structure of the hole-only devices was ITO/PEDOT:PSS/donor/Au. The J^1/2^-V curves were measured as shown in Supplementary Material. The relation of J and V could be described by J = 9ε_0_εμ(V_app_-V_s_-V_bi_)^2^/8L^3^, where J was the current density, ε_0_ was the permittivity of free space, ε was the relative permittivity of the p-type small molecules, μ was the hole mobility, V_app_ was the applied voltage, V_s_ was the voltage drop from series resistance of the substrate, V_bi_ was the built-in voltage and L was the thickness of the active layers (Qu et al., 2017). The hole mobilities were calculated with the fitted slope of the J^1/2^-V curves, which were 3.62^*^10^−6^ cm^2^ V^−1^ s^−1^ and 2.27^*^10^−5^ cm^2^ V^−1^ s^−1^ for **DTRDTQX** and **DTIDTQX**, respectively. The hole mobility of **DTIDTQX** was higher than that of **DTRDTQX**, which contributed to the decent photovoltaic performance of **DTIDTQX**-based OSCs. All the experimental data showed that **DTIDTQX** and **DTRDTQX** were promising donor candidates for small molecule OSCs and improved photovoltaic performance of OSCs based on **DTIDTQX** and **DTRDTQX** would be foreseen in the future.

## Conclusions

Two small molecules **DTRDTQX** and **DTIDTQX** with the D-A-A structure were studied in this work. **DTRDTQX** and **DTIDTQX** were used as the donors in bulk-heterojunction solar cells. The optimal OSCs based on **DTRDTQX**:C_70_(1:2) and **DTIDTQX**:C_70_(1:2) were achieved with the PCE of 1.44% and 1.70%, respectively. The photovoltaic properties of **DTIDTQX** were superior to those of **DTRDTQX**, which was attributed to the narrow band gap (1.37 eV) and the high hole mobility (2.27^*^10^−5^ cm^2^ V^−1^ s^−1^) of **DTIDTQX**. Therefore, **DTRDTQX** and **DTIDTQX** would be promising donor materials for organic solar cells in future.

## Author contributions

All authors listed have made a substantial, direct and intellectual contribution to the work, and approved it for publication.

### Conflict of interest statement

The authors declare that the research was conducted in the absence of any commercial or financial relationships that could be construed as a potential conflict of interest. The reviewer XZ and handling Editor declared their shared affiliation.
