# Supplementary material for: Two Novel Small Molecule Donors and the Applications in Bulk-Heterojunction Solar Cells
Source: Front Chem. 2018 Jul 2;6:260. doi: 10.3389/fchem.2018.00260 (PMC6036481; doi:10.3389/fchem.2018.00260)
Supplement: Supplementary file 1 [file Data_Sheet_1.DOC]

Supplementary Material

# Two Novel Small Molecule Donors and the Applications in Bulk-Heterojunction Solar Cells

Xin Qi, Yuan-Chih Lo, Yifan Zhao, Liyang Xuan, Hao-Chun Ting, Ken-Tsung Wong*, Mostafizur Rahaman, Zhijian Chen and Bo Qu*

*** Correspondence:** Bo Qu: bqu@pku.edu.cn; Ken-Tsung Wong, kenwong@ntu.edu.tw

**Synthesis**

*Synthesis of 5-bromo-8-methylquinoxaline* (**5**)

Added **3** (10 g, 43.65 mmol) into a mixture of EtOH (300 mL) and 12M HCl (73 mL) and then heated to reflux temperature. Then with stirring, Fe-powder (24.37 g, 452.63 mmol) were add into the solution in small portions. After heating 2 hours, the reaction solution was cooled down to room temperature. Then a water solution of NaOH (88 g) and EDTA (160 g) were added into the reaction and stirred for 30 mins. After that, 10% NaOH_(aq)_ was added for alkalization to make sure the pH > 10. The reaction mixture was then extracted with dichloromethane (DCM). The organic layer was then washed with water and brine and dried by MgSO_4_. The organic solvent was removed by rotary evaporation affording **4** (8.15g) as yellow oil. Took some of **4** (1 g, 5 mmol) and mixed with Glyoxal (40 wt%, 1.15 mL, 10 mmol), ethanol (30 mL) and few drops of triethylamine. The mixture was stirred at room temperature overnight. The precipitate was collected by filtration and purified by column chromatography in silica gel with EA/hexane (Hex) (v/v, 1/5) as eluent, obtaining **5** as white solid (680 mg, 61%). M.p. 148-150 °C; ^1^H NMR (CDCl_3_, 400 MHz) δ 8.95 (d, *J* = 1.6 Hz, 1H), 8.89 (d, *J* = 2.0 Hz, 1H), 7.99 (d, *J* = 7.6 Hz, 1H), 7.50 (dd, *J* = 0.8, 7.6 Hz, 1H), 2.77 (d, *J* = 0.8 Hz, 3H); ^13^C NMR (CDCl_3_, 100 MHz) δ 144.8, 144.1, 143.0, 140.5, 137.8, 133.1, 130.2, 121.1, 17.3; HRMS (m/z, FAB^+^) calcd for C_9_H_7_^79^BrN_2_: 221.9794, found: 221.9794, calcd for C_9_H_7_^81^BrN_2_: 223.9772, found: 223.9771; IR (KBr): ν 405, 416, 421, 429, 436, 440, 444, 451, 459, 484, 515, 545, 581, 621, 697, 811, 822, 883, 919, 978, 1003, 1024, 1076, 1092, 1114, 1195, 1214, 1335, 1380, 1397, 1458, 1488, 1563, 1598, 1861, 2918 cm^-1^.

*Synthesis of 5-bromo-8-(dibromomethyl)quinoxaline* (**6**)

A mixture of **5** (680 mg, 3.05 mmol), azobisisobutylnitrile (100 mg, 0.91 mmol), and *N*-bromosuccinimide (1.62 g, 9.15 mmol) in chlorobenzene (8 mL) was heated to 100 °C with stirring for 3 hours. After been cooled down to room temperature, the reaction mixture was filtered to remove precipitate. The filtrate was then washed with water and brine, and then the organic layer was dried by MgSO_4_. The organic solvent was removed by rotary evaporation, and the crude compound was purified by column chromatography in silica gel with DCM/Hex (v/v, 2/1) as eluent, obtaining **6** as white solid (809 mg, 69.6%). M.p. 111-113 °C; ^1^H NMR (CDCl_3_, 400 MHz) δ 9.02 (d, *J* = 1.6 Hz, 1H), 8.93 (d, *J* = 2.0 Hz, 1H), 8.30~8.28 (m, 1H), 8.21~8.18 (m, 1H); ^13^C NMR (CDCl_3_, 100 MHz) δ 145.8, 144.9, 139.7, 139.6, 138.1, 133.6, 131.2, 125.8, 34.5; HRMS (m/z, FAB^+^) calcd for C_9_H_5_^79^Br_3_N_2_: 377.8003, found: 377.8008, calcd for C_9_H_5_^81^Br_3_N_2_: 383.7941, found: 383.7940, calcd for C_9_H_5_^79^Br_2_^81^BrN_2_: 379.7982, found: 379.7991, calcd for C_9_H_5_^79^Br^81^Br _2_N_2_: 381.7962, found: 381.7968; IR (KBr): ν 401, 405, 413, 421, 428, 432, 436, 440, 443, 448, 451 , 455, 463, 530, 555, 566, 621, 683, 730, 778, 818, 849, 870, 938, 960, 1012, 1037, 1067, 1124, 1160, 1198, 1239, 1281, 1326, 1375, 1454, 1480, 1558, 1699, 1917, 3029, 3069 cm^-1^.

*Synthesis of 8-bromoquinoxaline-5-carbaldehyde* (**7**)

A mixture of **6** (460 mg, 1.21 mmol), calcium carbonate (400 mg, 4 mmol) dissolved in 10 mL of water and acetonitrile (20 mL) was stirred and heated up to reflux temperature for 8 hours. After the reaction was cooled down to room temperature, the reaction mixture was then filtered to remove participate and washed with DCM to make sure the entire product has been recovered. The filtrate was extracted with DCM and washed with water and brine. The organic layer was dried by MgSO_4_ and the organic solvent was removed by rotary evaporation, and the crude compound was purified by column chromatography in silica gel with DCM/Hex (v/v, 2/1) as eluent, obtaining **7** as white solid (262 mg, 91.3%). M.p. 193-195 °C; ^1^H NMR (CDCl_3_, 400MHz) δ 11.31 (s,1 H), 9.07 (d, *J* = 2.0 Hz, 1H), 9.04 (d, *J* = 2.0 Hz, 1H), 8.27 (q, *J* = 5.9 Hz, 2H); ^13^C NMR (CDCl_3_, 100MHz) δ 190.1, 146.1, 145.8, 142.9, 140.0, 133.4, 131.7, 131.4, 129.7; HRMS (m/z, FAB^+^) calcd for C_9_H_5_^79^BrN_2_O: 235.9585, found: 235.9586, calcd for C_9_H_5_^81^BrN_2_O: 237.9565, found: 237.9575; IR (KBr): ν 402, 405, 413, 423, 432, 441, 454, 460, 474, 574, 611, 770, 840, 850, 880, 932, 1014, 1036, 1093, 1116, 1201, 1215, 1280, 1334, 1363, 1399, 1457, 1485, 1554, 1585, 1703, 1741, 1812, 1861, 1932, 1971, 2889, 3024, 3076 cm^-1^.

*Synthesis of 8-(5-(di-p-tolylamino)thiophen-2-yl)quinoxaline-5-carbaldehyde* (**9**)

To a mixture of **7** (1.50 g, 6.33 mmol), PdCl_2_(PPh_3_)_2_ (222 mg, 0.32 mmol) in anhydrous toluene (45 mL) under argon atmosphere, *N*,*N*-di-*p*-tolyl-5-(tri-n-butylstannyl) -thiophen-2-amine (**8**) (7.28 mmol) was added through syringe with stirring, and then heated at 110 °C for 2 hours. After the reaction mixture was cooled down to room temperature, the solvent was removed by rotary evaporation, and the crude compound was then purified by column chromatography in silica gel with DCM/Hex (v/v, 1/2) as eluent to obtain **9** (2.11 g, 4.91 mmol, 77.6%). M.p. 214~215 °C; ^1^H NMR (CDCl_3_, 400MHz) δ 11.34 (s, 1H), 8.97 (d, *J* = 2.0 Hz, 2H), 8.42 (d, *J* = 8.0 Hz, 1H), 7.94 (d, *J* = 7.6 Hz, 1H), 7.59 (d, *J* = 8.8 Hz, 2H), 7.13~7.11 (m, 10H),2.35 (s, 6H); ^13^C NMR (CDCl_3_, 100MHz) δ 190.2, 148.3, 146.6, 144.6, 144.2, 142.7, 133.0, 131.1, 129.8, 129.6, 129.1, 129.0, 128.9, 125.1, 121.6, 120.2, 21.3; HRMS (m/z, FAB^+^) calcd for C_29_H_23_N_3_O: 429.1841, found: 429.1838; IR (KBr) ν 401, 405, 409, 413, 417, 421, 424, 428, 432, 436, 440, 444, 447, 455, 477, 500, 511, 518, 562, 587, 60, 622, 644, 670, 705, 723, 779, 808, 822, 831, 844, 867, 936, 961, 1016, 1038, 1102, 1181, 1193, 1213, 1269, 1299, 1307, 1324, 1357, 1376, 1406, 1427, 1468, 1488, 1507, 1570, 1599, 1682, 1901, 2858, 2919, 3027 cm^-1^.

*Synthesis of 2-((8-(5-(di-p-tolylamino)thiophen-2-yl)quinoxalin-5-yl)methylene)-1H-indene-1,3(2H)-dione* (**DTIDTQX**)

To a mixture of **10** (1.00 g, 2.23 mmol) and 1,3-indanedione (504 mg, 3.45 mmol) in 60 mL of chloroform, 10 drops of trimethylamine was added dropwise with stirring under argon atmosphere. Then the mixture solution was heated up to reflux for 2 hours. Then the solvent was removed through the rotary evaporation. The crude product was purified by column chromatography on silica gel with DCM/Hex (v/v, 3/1) as eluent to obtain **DTIDTQX** as black solid (0.590 g, 1.05 mmol, 46.9 %). M.p. 263.3 °C ^1^H NMR (CDCl_3_ , 400 MHz) δ 7.58-7.59 (m, 6H), 7.50 (d, J=6.4 Hz, 2H), 7.42-7.44 (m, 4H), 7-14-7.17 (m, 4H), 6.27 (d, J=6.4 Hz, 2H). ^13^C NMR (126 MHz, CDCl_3_) δ 190.54, 189.81, 160.31, 144.84, 144.56, 143.55, 143.07, 142.58, 140.67, 140.24, 139.18, 138.67, 135.71, 135.03, 134.97, 134.16, 130.05, 129.38, 128.61, 127.35, 127.09, 124.40, 124.37, 123.17, 123.10, 115.29, 20.92. HRMS (m/z, MALDI, CHCA) calcd for C_36_H_25_N_3_O_2_S : 563.166, found: 563.169.

*Synthesis of (Z)-5-((8-(5-(di-p-tolylamino)thiophen-2-yl)quinoxalin-5-yl)methylene)-3-ethyl-2-thioxothiazolidin-4-one* (**DTRDTQX**)

To a mixture of **10** (500 mg, 1.15 mmol) and 3-ethylrhodanine (278 mg, 1.73 mmol) in 45 mL of DCM, 10 drops of trimethylamine was added dropwise with stirring under argon atmosphere. Then the mixture solution was heated up to reflux for 27 hours. Then the solvent was removed through the rotary evaporation. The crude product was purified by column chromatography on silica gel with DCM/Hex (v/v, 1/1) as eluent to obtain **DTRDTQX** as black solid (228 mg, 0.387 mmol, 33.6 %) ^1^H NMR (CDCl_3_ , 400 MHz) δ 7.58-7.59 (m, 6H), 7.50 (d, J=6.4 Hz, 2H), 7.42-7.44 (m, 4H), 7-14-7.17 (m, 4H), 6.27 (d, J=6.4 Hz, 2H). ^13^C NMR (126 MHz, CDCl_3_) δ 194.21, 167.65, 159.08, 144.99, 143.83, 143.68, 141.89, 139.11, 136.40, 133.91, 131.73, 130.01, 128.70, 128.61, 128.50, 127.21, 125.01, 124.14, 123.97, 115.76, 77.26, 77.00, 76.75, 39.81, 20.90, 12.31. HRMS (m/z, MALDI, CHCA) calcd for C_32_H_26_N_4_OS_3_ : 578.126, found: 578.129.


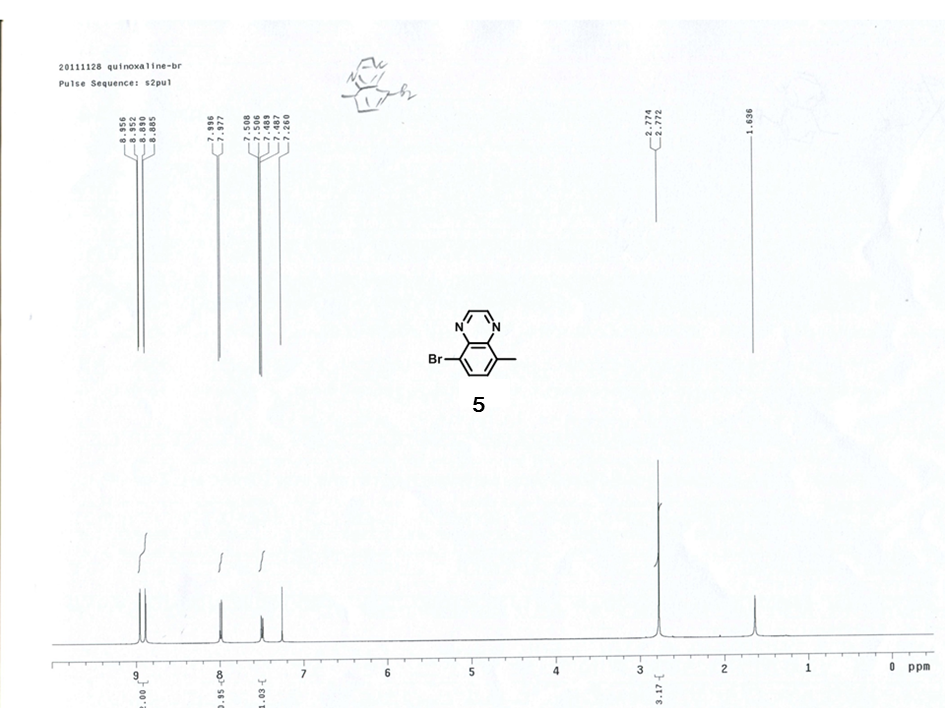


^1^H NMR of **5**


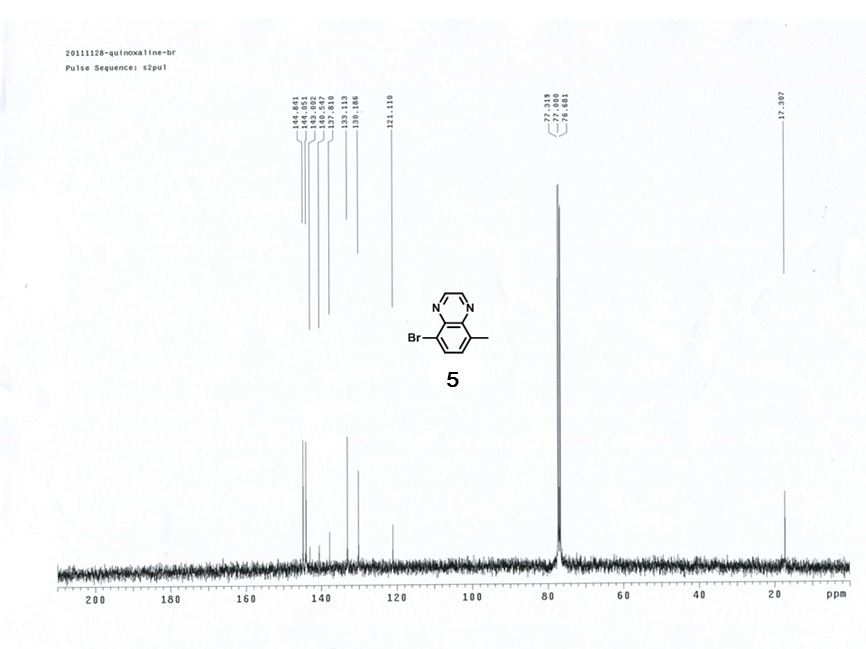


^13^C NMR of **5**


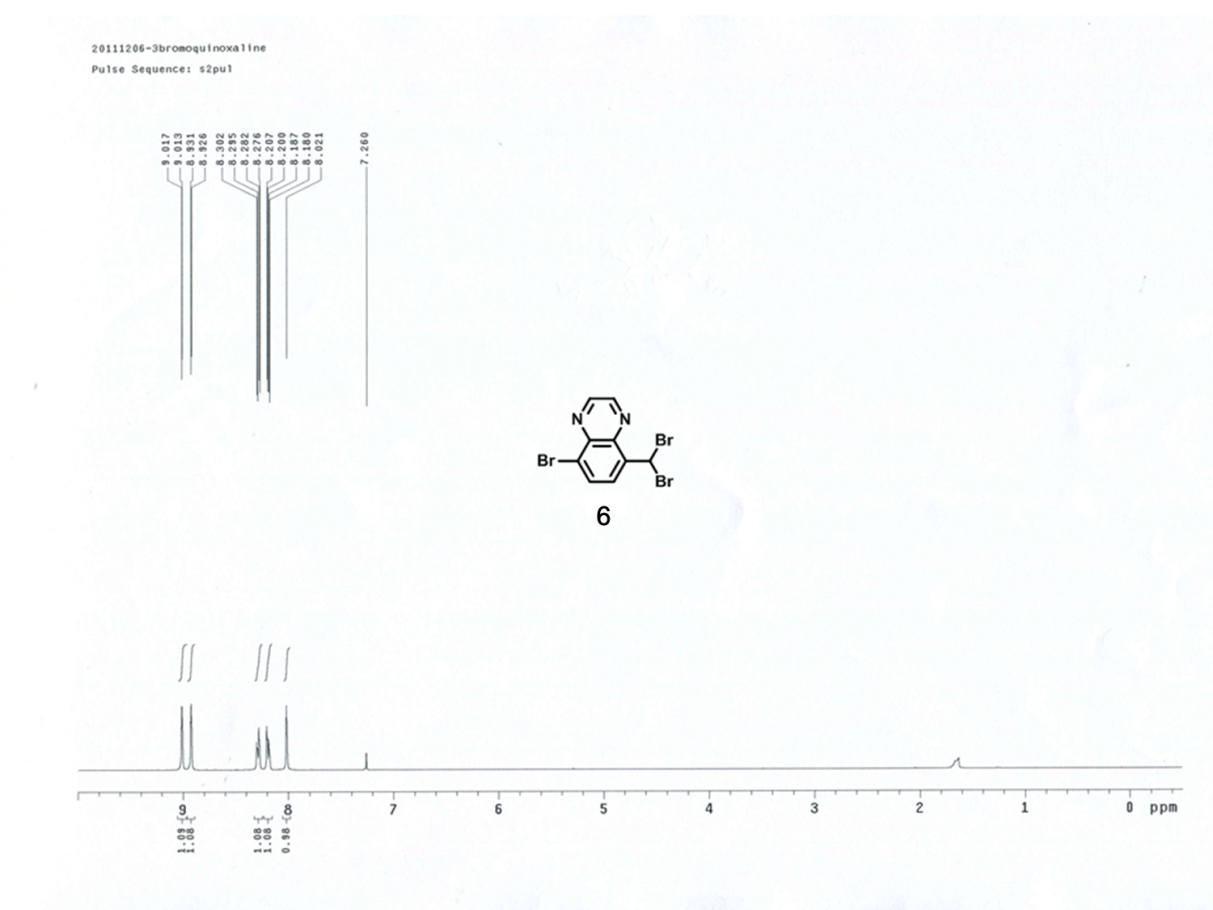


^1^H NMR of **6**


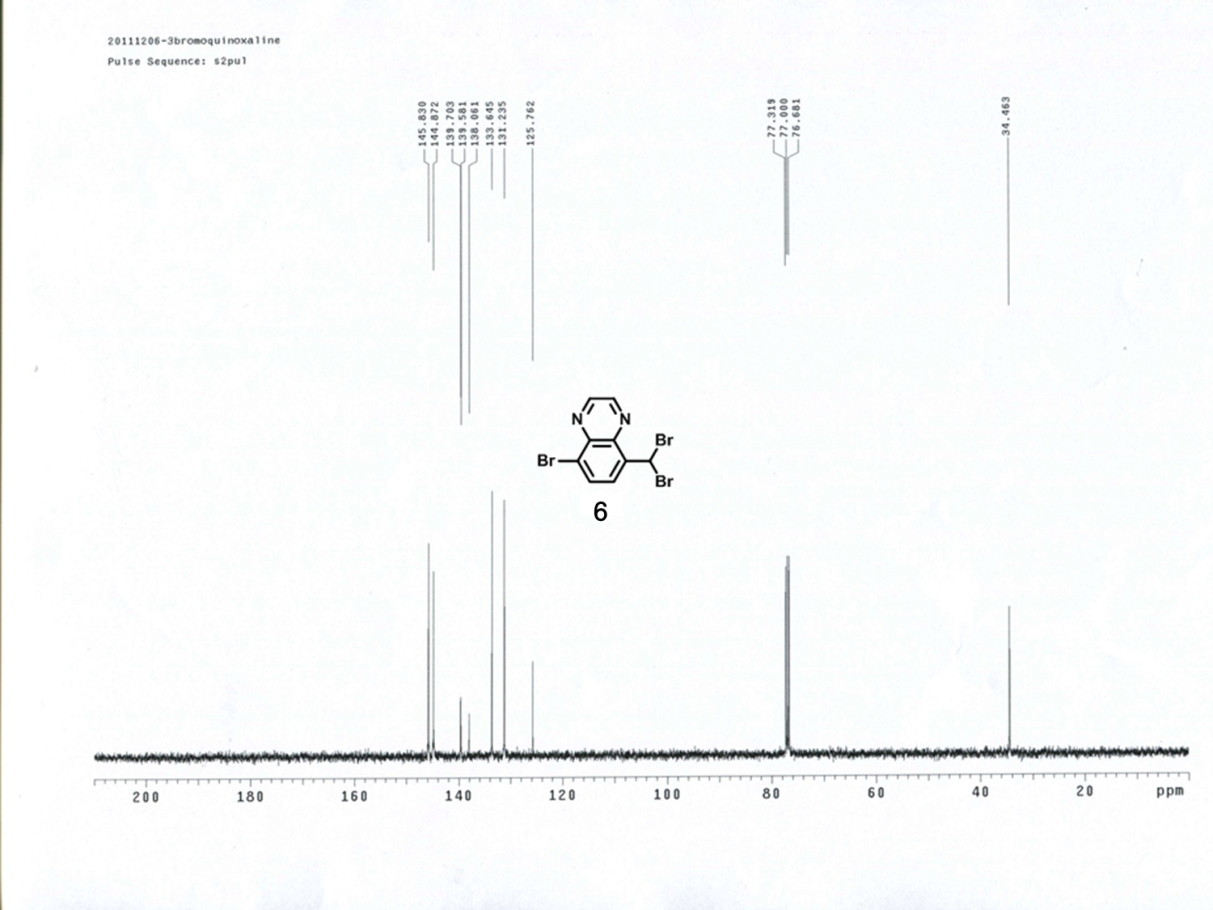


^13^C NMR of **6**


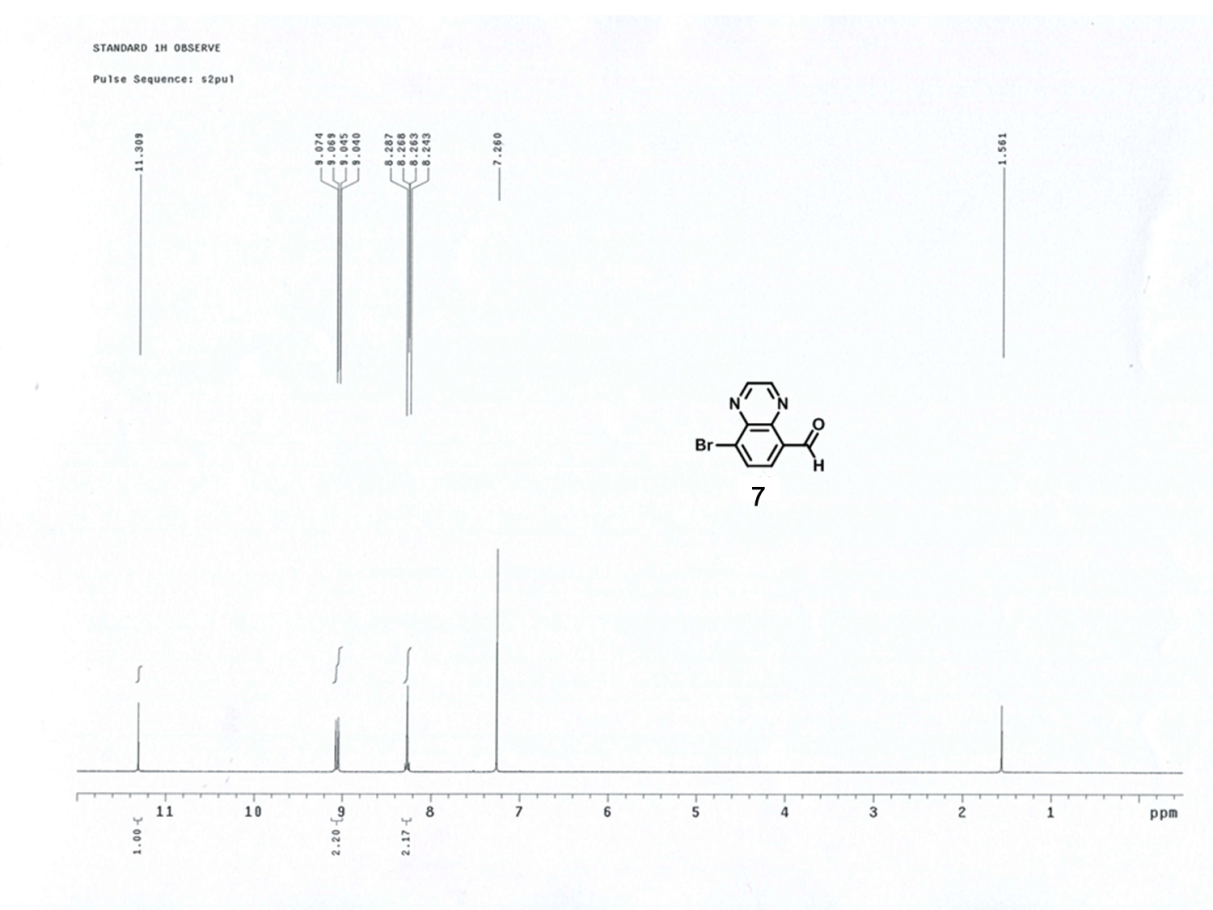


^1^H NMR of **7**


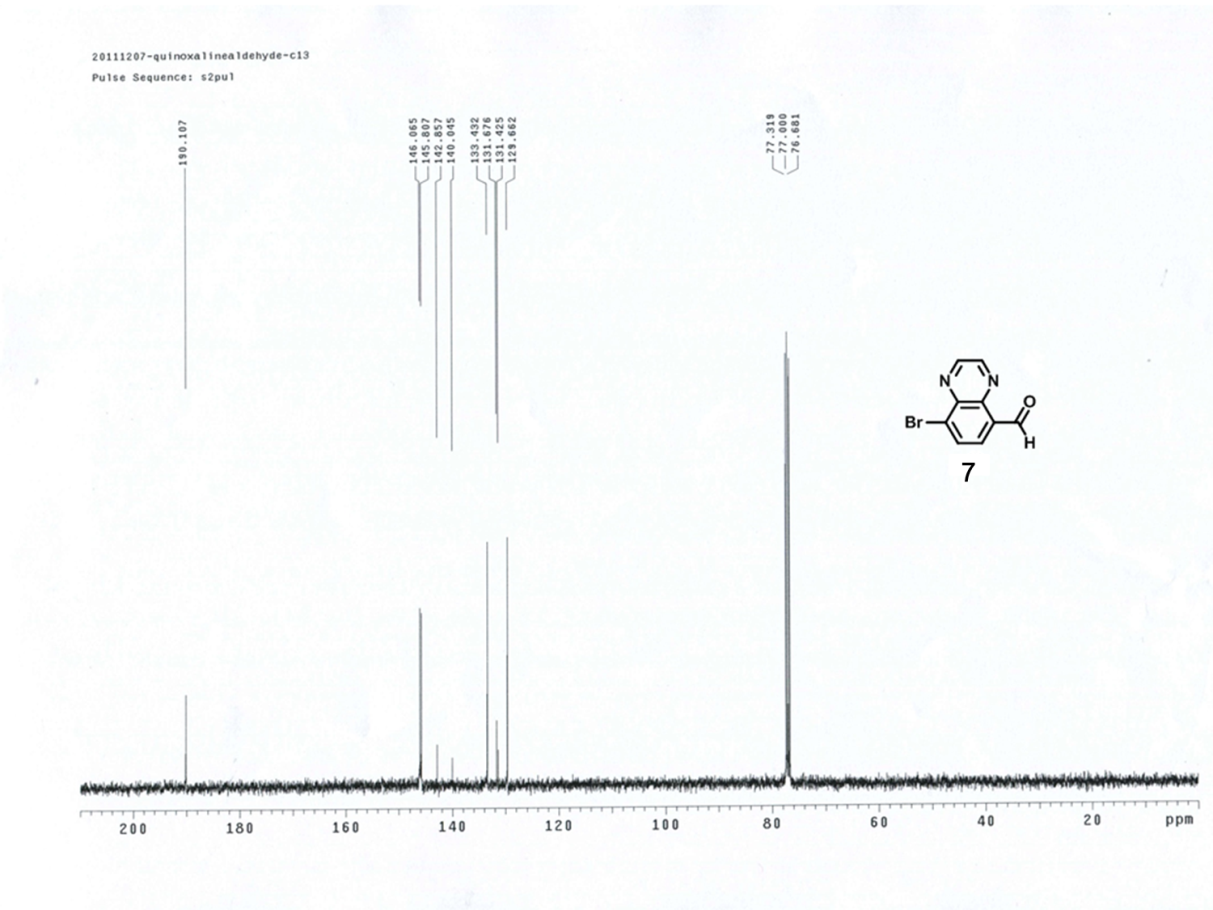


^13^C NMR of **7**


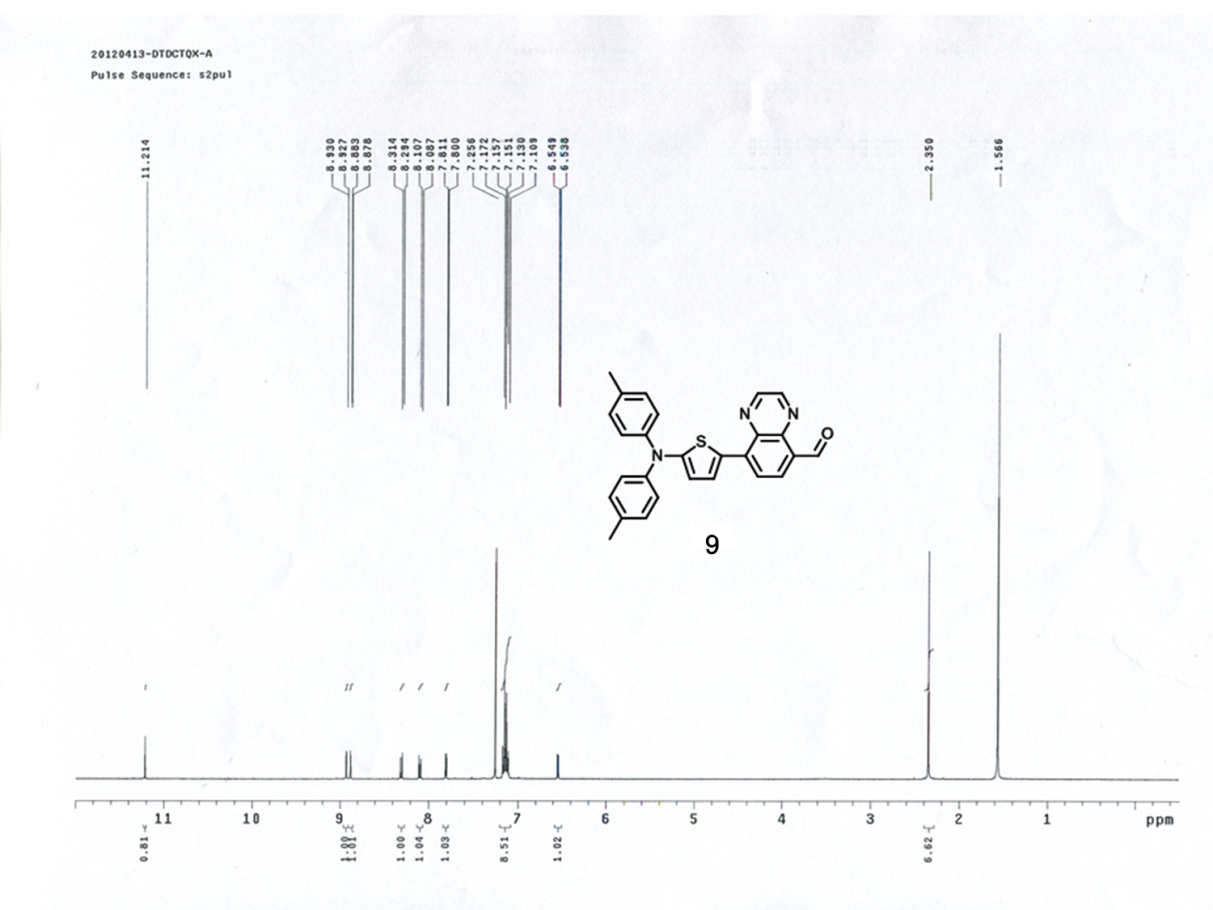


^1^H NMR of **9**


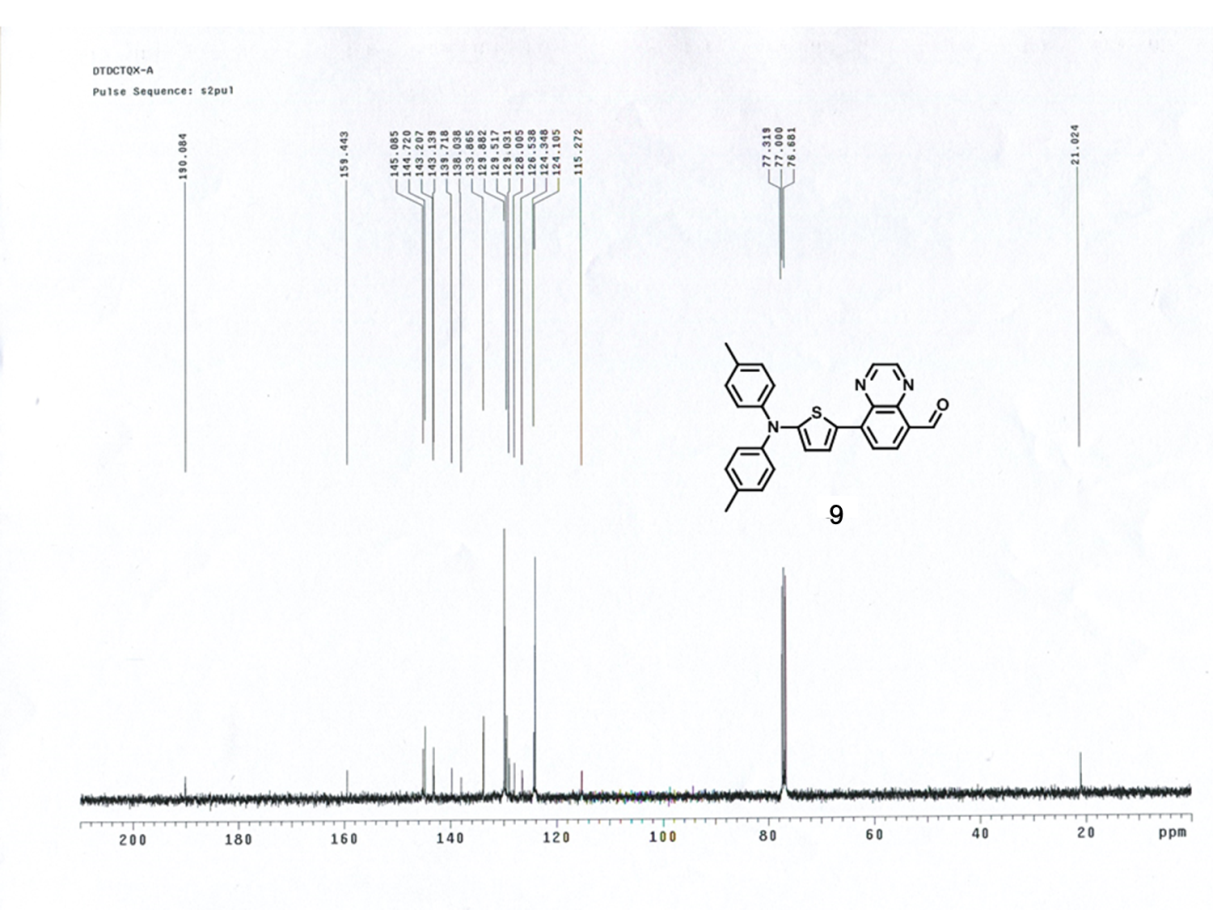


^13^C NMR of **9**


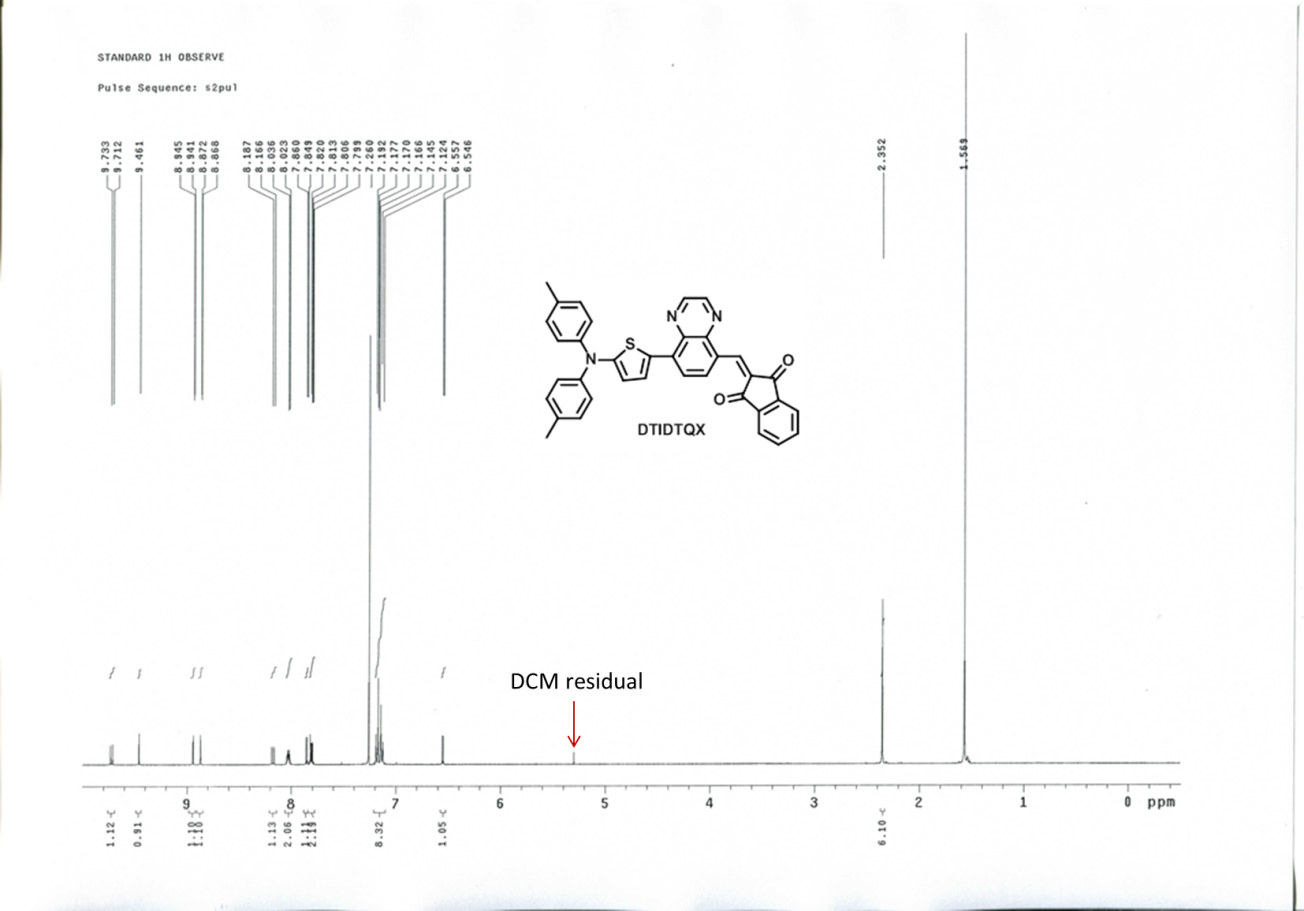


^1^H NMR of **DTIDTQX**


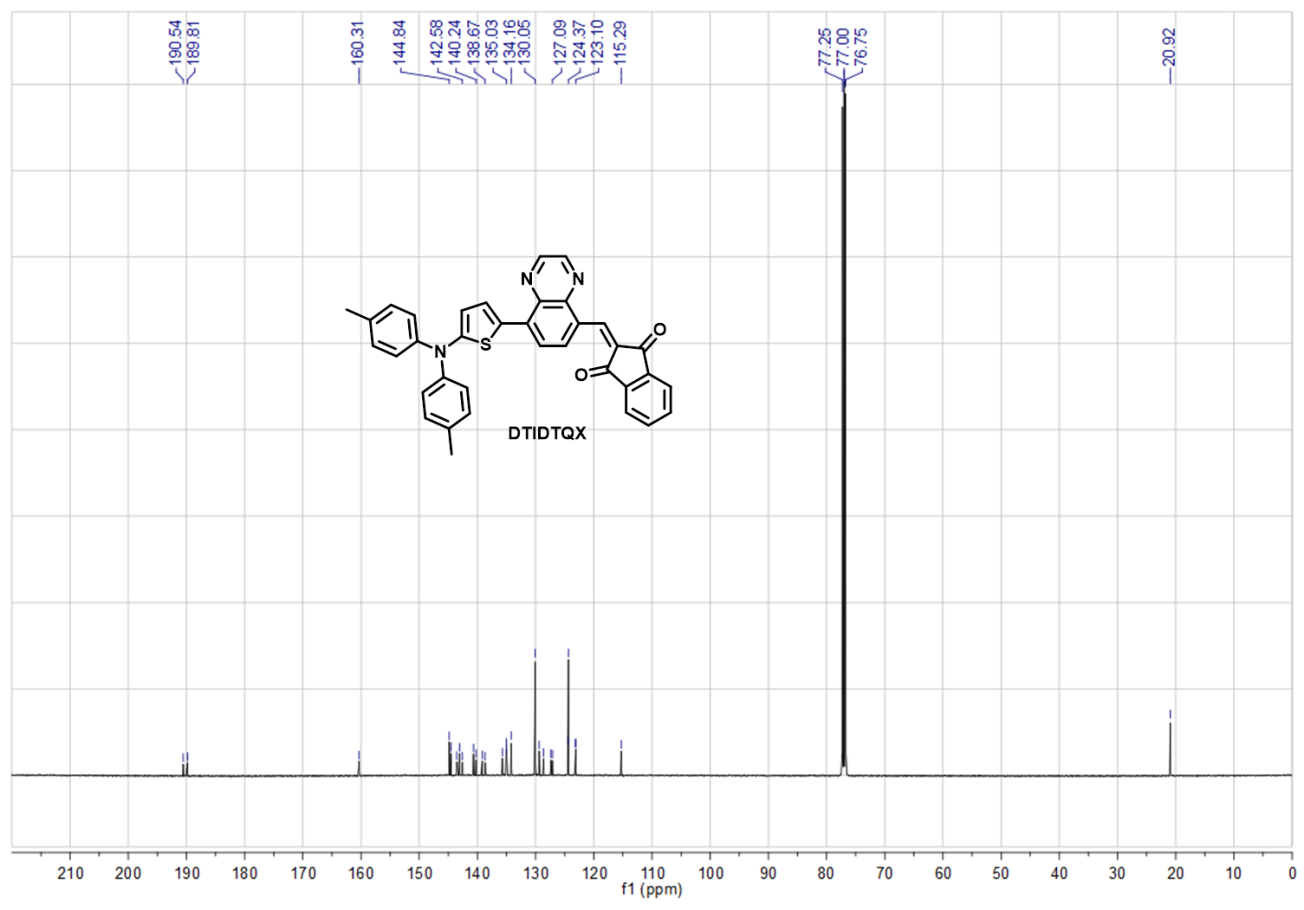


^13^C NMR of **DTIDTQX**


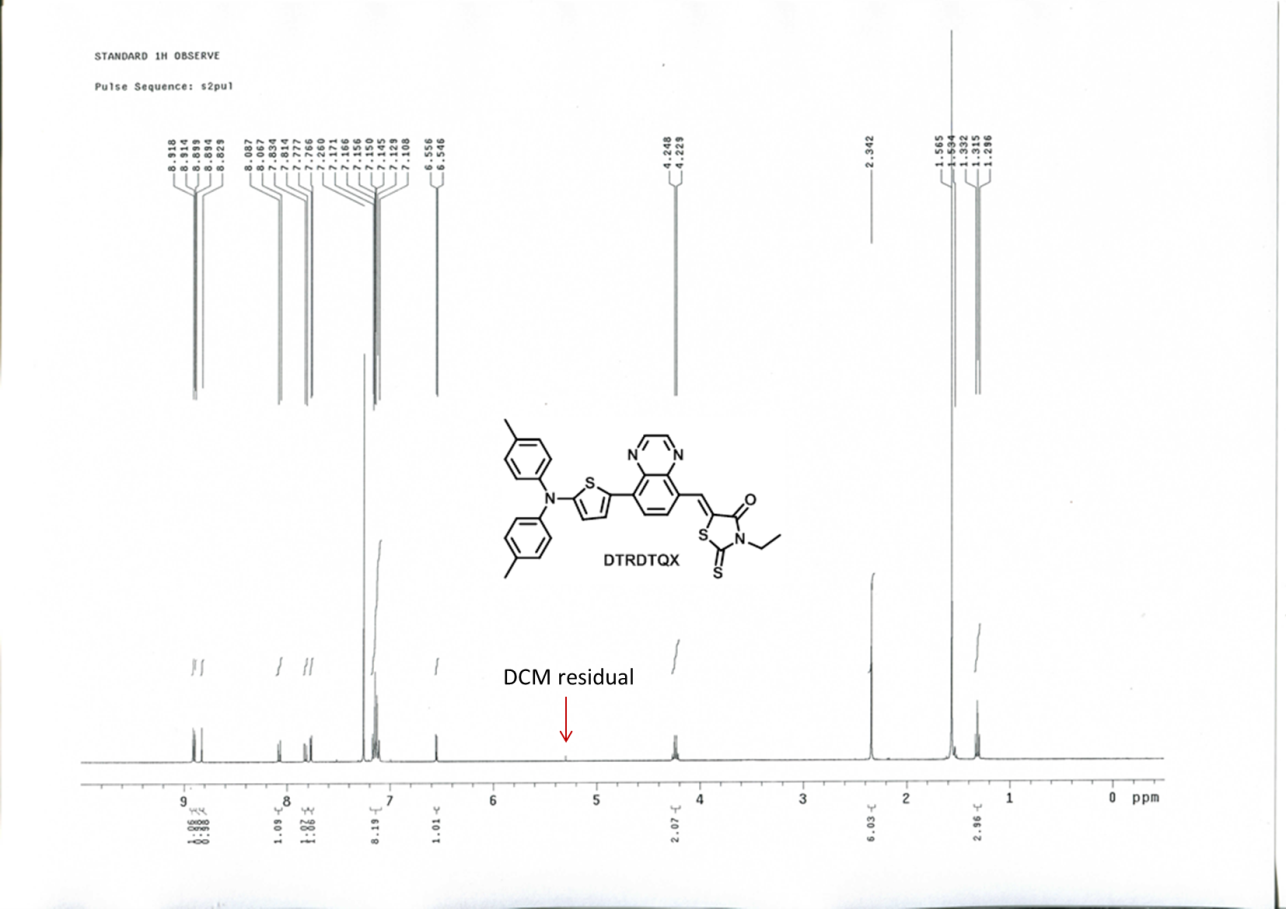


^1^H NMR of **DTRDTQX**


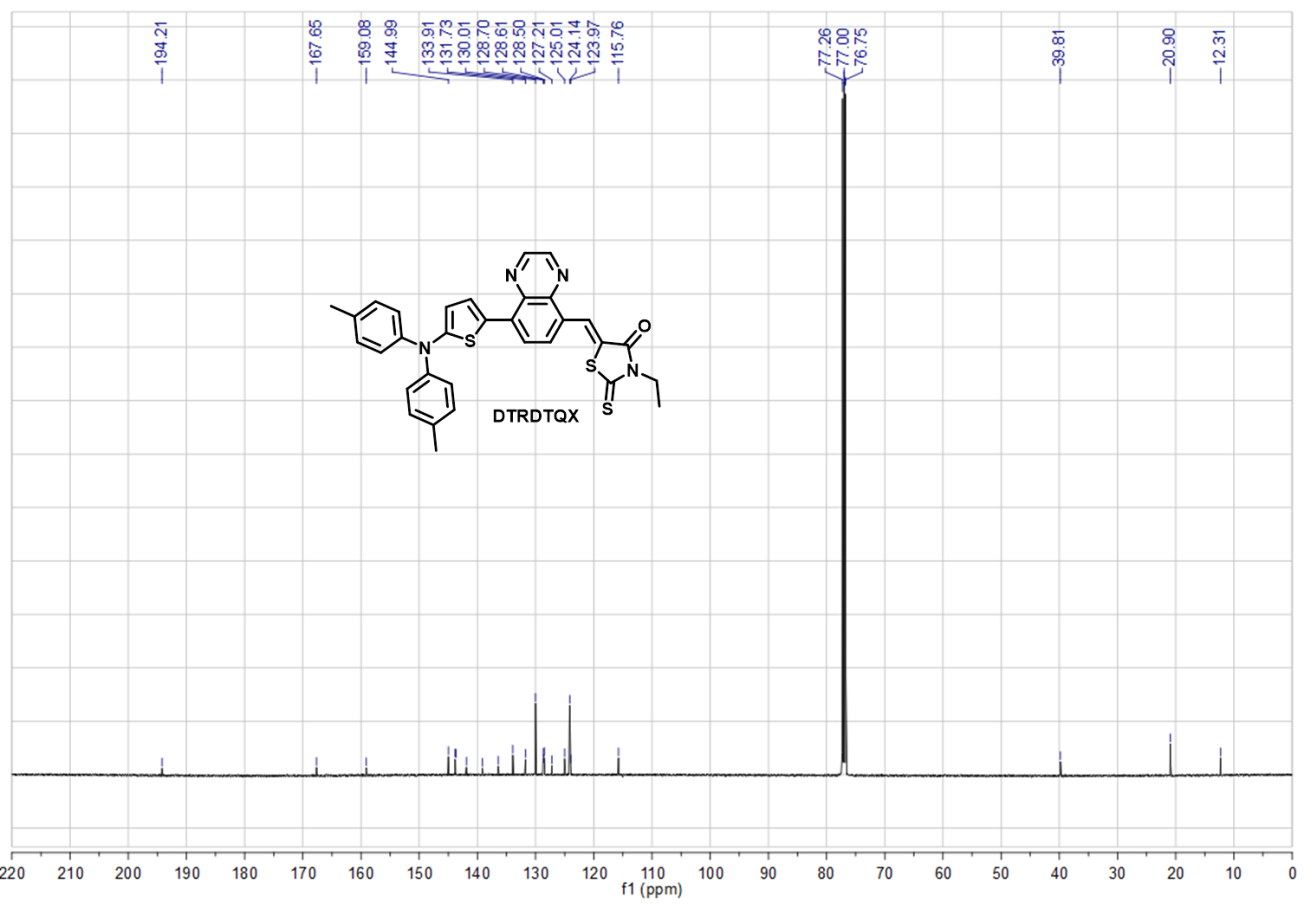


^13^C NMR of **DTRDTQX**


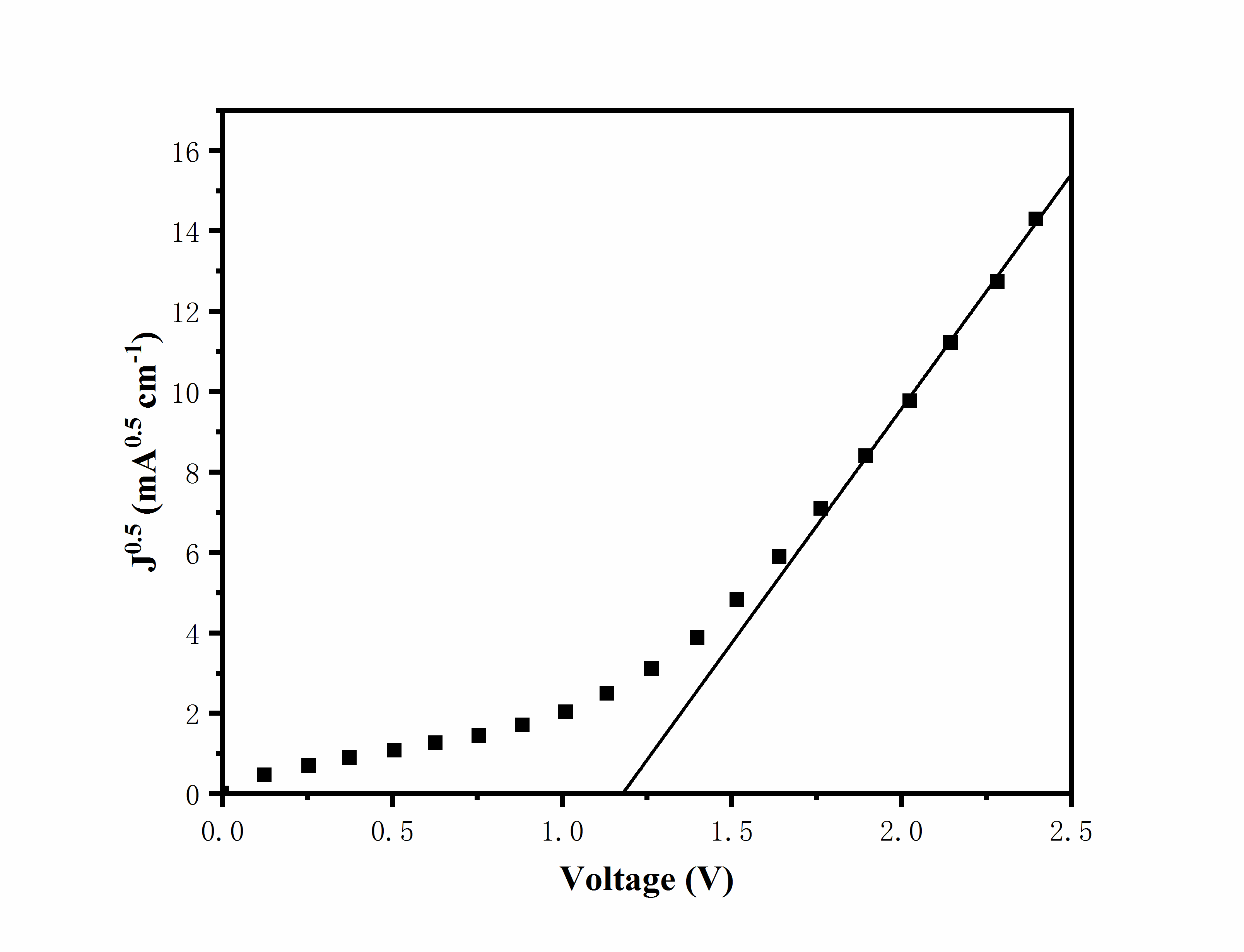


J^1/2^–V curves for DTRDTQX, L=18nm


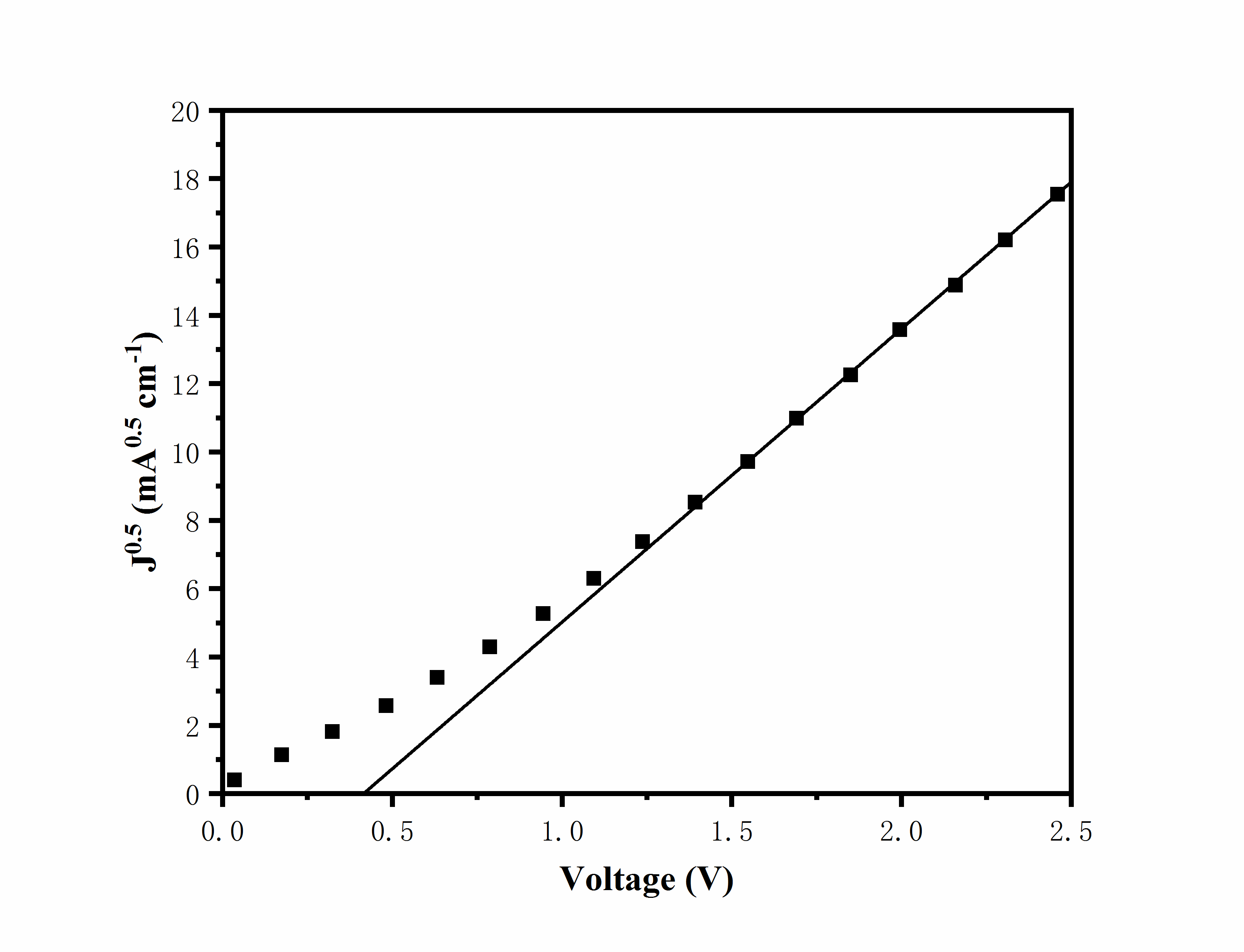


J^1/2^–V curves for DTIDTQX, L=41nm
